# Pinning Down the Transcription: A Role for Peptidyl-Prolyl *cis-trans* Isomerase Pin1 in Gene Expression

**DOI:** 10.3389/fcell.2020.00179

**Published:** 2020-03-20

**Authors:** Xiangming Hu, Lin-Feng Chen

**Affiliations:** ^1^Fujian Key Laboratory for Translational Research in Cancer and Neurodegenerative Diseases, Institute for Translational Medicine, School of Basic Medical Sciences, Fujian Medical University, Fuzhou, China; ^2^Department of Biochemistry, University of Illinois at Urbana-Champaign, Urbana, IL, United States; ^3^Carl R. Woese Institute for Genomic Biology, University of Illinois at Urbana-Champaign, Urbana, IL, United States

**Keywords:** conformational change, isomerization, phosphorylation, Pin1, transcription, RNA polymerase II, transcription factor

## Abstract

Pin1 is a peptidyl-prolyl *cis-trans* isomerase that specifically binds to a phosphorylated serine or threonine residue preceding a proline (pSer/Thr-Pro) motif and catalyzes the *cis-trans* isomerization of proline imidic peptide bond, resulting in conformational change of its substrates. Pin1 regulates many biological processes and is also involved in the development of human diseases, like cancer and neurological diseases. Many Pin1 substrates are transcription factors and transcription regulators, including RNA polymerase II (RNAPII) and factors associated with transcription initiation, elongation, termination and post-transcription mRNA decay. By changing the stability, subcellular localization, protein-protein or protein-DNA/RNA interactions of these transcription related proteins, Pin1 modulates the transcription of many genes related to cell proliferation, differentiation, apoptosis and immune response. Here, we will discuss how Pin regulates the properties of these transcription relevant factors for effective gene expression and how Pin1-mediated transcription contributes to the diverse pathophysiological functions of Pin1.

## Introduction

Prolyl isomerases (PPIases) catalyze the *cis-trans* isomerization of the peptidy prolyl (X-Pro) bonds. There are three distinct families of PPIases: cyclophilins (CyPs), FK506-binding proteins (FKBPs), and parvulins (Zhou and Lu, 2016). Pin1 belongs to the parvulin family and is comprised of an N-terminal WW domain serving as a phosphoprotein-binding module and a C-terminal catalytic domain that is distinct from other conventional PPIases (Zhou and Lu, 2016). Because of its unique WW and PPIase domains, Pin1 specifically isomerizes the pSer/Thr-Pro motif and regulates the functions of a defined group of phosphoproteins by altering their conformations (Liou et al., 2011). Pin1-mediated post-phosphorylation regulation has profound effects on multiple cellular and biological processes, including cell cycle, cell differentiation and death, and metabolic and immune response (Liou et al., 2011; Zhou and Lu, 2016). Aberrant expression of Pin1 has been identified to be associated with many diseases, especially in cancer and neurodegenerative disorders, such as Parkinson’s disease (PD) and Alzheimer’s disease (AD) (Liou et al., 2011; Zhou and Lu, 2016). While Pin1 is highly expressed in the majority of cancers and promotes cancer progression, its expression is down-regulated in neurodegenerative diseases (Liou et al., 2011; Zhou and Lu, 2016), highlighting the diverse regulatory functions of Pin1 in physiology and diseases.

The temporal and spatial eukaryotic gene expression is a highly orchestrated molecular event that is regulated at multiple levels and is responsible for the distinct cellular responses and functions. The multi-level regulation includes the signal-dependent activation of tissue-specific transcription factors, the remodeling of chromatin on promoters and enhancers, the pausing and release of RNAPII, the post-transcriptional processing of mRNA, and the translational regulation (Splinter and De Laat, 2011; Dong et al., 2012; Hensel and Xiao, 2013). Post-translational modifications, especially phosphorylation, play important roles in the multi-level regulation of gene expression (Pawson and Scott, 2005). Many transcription factors and transcription related proteins undergo phosphorylation and activate gene expression in response to intra- and extracellular stimuli (Hunter and Karin, 1992). The reversible phosphorylation on serine or threonine residues preceding a proline (pSer/Thr-Pro) has emerged as a pivotal switch for controlling the activities of participating transcription components in gene expression (Shaw, 2007; Hanes, 2015). Pin1’s ability to regulate many cellular processes might rely on its ability to regulate the expression of various genes by binding to the phosphorylated transcription regulators. More than 40 different kinds of transcription related proteins, including transcription activators and general transcription machinery components, have been identified to be Pin1 substrates (Table 1). Pin1 binds to the pSer/Thr-Pro motifs of these proteins and regulates the gene transcription by altering the stability, subcellular localization, protein-protein interactions, and protein-DNA/RNA interaction of these factors (Xu and Manley, 2007a). In this review, we summarize how Pin1 controls the activity of these transcription regulators for the spatiotemporal expression of genes involved in cell cycle, cell proliferation and growth, metabolism and inflammation, thus contributing to the diverse functions of Pin1 in physiology and disease.

**TABLE 1:** List of Pin1 substrates in transcription regulation.

Substrates	Motif	Regulation by Pin1	Cellular consequence of Pin1 interaction	Evidence of isomerization	References
**Transcription factors**					
**(1) Nucleocytoplasmic shuttling**					
RelA	T254	Increased nuclear accumulation and stability	Cell survival, proliferation and inflammation	Yes	Ryo et al., 2003; Atkinson et al., 2009; Fan et al., 2009; Shinoda et al., 2015
β-catenin	S246	Increased nuclear accumulation and stability	Cancer cell proliferation, osteogenesis	Yes	Ryo et al., 2001; Nakamura et al., 2012
Fox04	N/A	Deubiquitylation and decreased nuclear accumulation	Cell cycle and cancer cell proliferation	Yes	Brenkman et al., 2008
NFAT	N/A	Decreased nuclear accumulation	T cell activation	N/A	Liu et al., 2001
**(2) Protein stability**					
p53	S33, S46, T81, S315	Increased stability and transactivation	DNA damage response, cancer cell cycle arrest and apoptosis	Yes	Wulf et al., 2002; Zacchi et al., 2002; Zheng et al., 2002
p63	T538	Increased or decreased stability	Cancer and limb development	N/A	Li et al., 2013; Restelli et al., 2014
p73	S412, T442, T482	Increased stability and transactivation	Apoptosis	Yes	Mantovani et al., 2004
c-Jun	S63, S73	Increased stability	Ras and JNK signaling	Yes	Wulf et al., 2001; Pulikkan et al., 2010
Naong	S52, S65, S71, T287	Increased stability	Stem cell pluripotency	Yes	Moretto-Zita et al., 2010
Oct4	S12	Increased stability	Stem cell pluripotency	Yes	Nishi et al., 2011
FoxMl	S331, S704	Increase stability	Drug resistance	N/A	Kruiswijk et al., 2016; Wang et al., 2016
Osterix	S76, S80	Increase stability and transactivation	Osteogenic differentiation	Yes	Jang et al., 2005
ATF1	T184	Increase stability and transactivation	NPC tumorigenesis	N/A	Huang et al., 2016
TR3	S95, S140, S431	Increase stability and transactivation	Mitogenesis	Yes	Chen et al., 2012
Runx2	T408, T449, S472, S510	Increase sub-nuclear area accumulation and stability	Skeletal development, Osteoblast differentiation.	Yes	Lee et al., 2013; Yoon et al., 2013
Runx3	T209, T212, T231, S214	Degradation, suppresses transactivation	Breast cancer cell proliferation	Yes	Nicole Tsang et al., 2013
Smad3	T179, S204, S208, S213	Decreased stability	Cell migration and invasion	Yes	Nakano et al., 2009
IRF3	S339	Decreased stability	Antiviral responses	Yes	Saitoh et al., 2006
RAR	S77	Decreased stability	Cancer cell proliferation	Yes	Gianni et al., 2009
MEF2C	S98, S110	Decreased stability	Muscle terminal differentiation	Yes	Magli et al., 2010
Fox03	N/A	Decreased stability	Drug resistance	No	Shimizu et al., 2016
**(3) DNA binding activity and transcriptional activity**					
c-Myc	T58, S62	Decreased stability, increased DNA binding	Cancer cell proliferation	Yes	Yeh et al., 2004; Farrell et al., 2013
ERα	S118, S294	Increased dimerization, stability and transactivation activity	Cancer cell proliferation	Yes	Rajbhandari et al., 2014
HIFIα	S641, S643	Increase stability and transactivation	Angiogenesis	Yes	Jalouli et al., 2014; Han H. J. et al., 2016
SP1	T739	Increased stability, decreased DNA binding	Cell cycle progression	Yes	Yang et al., 2014
c-Fos	T232, T325, T331	Increased interaction with other transcription factors	Mitogen response	Yes	Monje et al., 2005
GR	S203, S211	Increased transactivation	Inflammatory response	Yes	Poolman et al., 2013
PPARγ	S273	Increased stability and transactivation	Adipogenesis	N/A	Fujimoto et al., 2010; Han Y. et al., 2016
Nur77	S152	Increased DNA binding and transactivation	Vascular disease and metabolism	No	van Tiel et al., 2012
Stat3	S272	Increased DNA binding and transactivation	EMT and type 2 diabetes	No	Lufei et al., 2007; Lv et al., 2013; Nakada et al., 2019
**Transcription cofactors**					
SRC-3	multiple sites	Increased interaction with p300 and degradation	Brease cancer cell proliferation	Yes	Yi et al., 2005
Notch1	S2122, T2133, S2137	Enhanced Notch1 cleavage and transcriptional activity	Notch signaling	Yes	Rustighi et al., 2009
SMRT	S1241, S1445, S1469	Decreased stability	Cancer cell proliferation and response to tamoxifen	Yes	Stanya et al., 2008
CRTC2	S136	Decreased nuclear accumulation	Glucose metabolism	N/A	Nakatsu et al., 2010
PRDM16	N/A	Decreased stability	Thermogenesis	N/A	Chi and Cohen, 2016
**RNA polymerase**					
Rpbl	S2, S5 of CTD	Altered phosphorylation of CTD	Transcription	Yes	Xu et al., 2003; Zhang et al., 2012
**Histone**					
Histone HI	multiple sites	Increased dephosphorylation and binding to chromatin	Transcription	Yes	Raghuram et al., 2013
**Transcription elongation regulators**					
Spt5	multiple sites	Increased binding to transcription regulators	Transcription	N/A	Lavoie et al., 2001
Brd4	T204	Increased stability and transcriptional activity	Transcription and cancer	Yes	Hu et al., 2017
**mRNA decay factors**					
SLBP	T171	Increased dephosphorylation	Cell cycle	Yes	Krishnan et al., 2012
AUF1	S83	Decreased AUF1-mRNA interactions	Eosinophil survival, T cell activation, allergic inflammation	Yes	Shen et al., 2005; Esnault et al., 2006
KSRP	S181	Increased dephosphorylation and mRNA interaction	Hyperparathyroidism	N/A	Nechama et al., 2009
HuR	N/A	mRNA binding affinity	Transcription	N/A	Krishnan et al., 2014

## Pin1 and DNA Binding Transcription Factors

Eukaryotic gene expression is regulated by genomic enhancers and promoters that are recognized by various tissues specific DNA binding transcription factors (Patikoglou and Burley, 1997). Pin1 regulates the activities of a spectrum of transcription factors, many of which are involved in cancer cell proliferation and inflammatory response (Lu and Zhou, 2007).

### Nucleocytoplasmic Shuttling of Transcription Factors

A key regulatory step in transcription is the nucleocytoplasmic shuttling of transcription factors, which are synthesized in the cytoplasm and need to be transported into the nucleus, where they bind to the promoters or enhancers to activate gene expression in response to different intra- or extracellular stimuli (Cartwright and Helin, 2000). A number of studies have shown that Pin1 regulates the nucleocytoplasmic shuttling of transcription factors for the activation or inactivation of transcriptional response. For example, Pin1 promotes nuclear localization of RelA subunit of NF-κB (Ryo et al., 2003) and β-catenin (Ryo et al., 2001). Upon cytokine stimulation, Pin1 binds to the phosphorylated Thr254-Pro motif in RelA and increases the nuclear accumulation of RelA by inhibiting its binding to IκBα (Ryo et al., 2003). IκBα, the inhibitor of NF-κB, is known to sequester NF-κB in the cytoplasm by masking the nuclear localization signal (NLS) of NF-κB (Chen and Greene, 2004). Thr254 of RelA is in the proximity of Ser238, Asp243, and Arg253, three key amino acids involved in the IκBα binding (Jacobs and Harrison, 1998). Phosphorylation of Thr254 and the subsequent of binding of Pin1 likely change the conformation of RelA, therefore preventing its interaction with IκBα□ (Ryo et al., 2003). NF-κB is a master regulator of inflammatory response and is also a key player in the cancer cell development (Chen and Greene, 2004). By regulating the activation of NF-κB, Pin1 promotes tumor progression and inflammatory cytokine production (Ryo et al., 2003; Atkinson et al., 2009; Fan et al., 2009; Shinoda et al., 2015).

The nucleocytoplasmic shuttling of β-catenin is regulated by its association with APC (the adenomatous polyposis coli), which contains two active nuclear export sequences (NES) for the nuclear export of β-catenin (Henderson, 2000). Pin1 recognizes phosphorylated Ser246-Pro motif of β-catenin. Interestingly, the Ser246-Pro motif is next to the APC binding site (Ryo et al., 2001). Therefore, Pin1-mediated isomerization of the pSer246-Pro peptide bond in β-catenin would affect its binding to APC, leading to the accumulation of β-catenin in the nucleus and the up-regulation of its target genes, such as cyclin D1 and c-Myc (Ryo et al., 2001). Since aberrant accumulation of β-catenin contributes to abnormal development and tumorigenesis, Pin1 regulates many processes in development and tumor formation, including osteoblast and neuronal differentiation, cancer cell proliferation, and drug resistance, via affecting the transcriptional activity of β-catenin (Ryo et al., 2001; Nakamura et al., 2012; Shin et al., 2016; Wang et al., 2016).

In addition to stimulating the nuclear accumulation of NF-κB and β-catenin, Pin1 can also sequester transcription factors in the cytoplasm to inactivate the target gene expression. The nuclear localization and the transcriptional activity of FOXO4, a tumor suppressor preventing the accumulation of cellular damage due to oxidative stress, are regulated by its monoubiquitination (Van Der Horst et al., 2006). In response to oxidative stress, Pin1 binds to phosphorylated FOXO4 and increases USP7-mediated FOXO4 deubiquitination, resulting in the decreased monoubiquitination and the increased cytoplasmic accumulation. Ultimately, binding of Pin1 to FOXO4 decreases its transcriptional activity toward its target genes, including the cell cycle arrest gene *p27kip1* (Brenkman et al., 2008).

Another example for the Pin1-mediated nucleocytoplasmic shuttling of transcription factor is nuclear factor activated T cell (NFAT), which is essential for T cell activation (Liu et al., 2001). Upon T cell activation, intracellular calcium is increased and NFAT is subject to dephosphorylation by the calcium- and calmodulin (CaM)-dependent protein phosphatase calcineurin, triggering the translocation of NFAT into the nucleus where it binds to the promoter region of a number of cytokines and activates their transcription (Zhu and Mckeon, 2000). Pin1 has been reported to form a stable complex with the phosphorylated form of NFAT, which contains 3 Pin1 binding motifs (Liu et al., 2001). Therefore, by controlling the nucleocytoplasmic shuttling, Pin1 functions as a negative regulator of NFAT and T cell activation.

### Stability of Transcription Factors

Another major mechanism of Pin1-mediated transcription factor regulation is through ubiquitin-mediated protein degradation (Liou et al., 2011; Dilworth et al., 2012; Hanes, 2015). Pin1 can either increase or decrease the stability of transcription factors, depending on the functionality of these transcription factors.

The tumor suppressor p53 is a key transcription factor regulating cellular pathways such as DNA repair, cell cycle, apoptosis and senescence and is a pivotal gatekeeper against cancer onset and progression (Zilfou and Lowe, 2009). A key regulatory mechanism for the transactivation of p53 is the E3 ligase MDM2-mediated ubiquitination and degradation (Zilfou and Lowe, 2009; Nag et al., 2013). In response to DNA damage, p53 is stabilized by its release from MDM2 and activates its downstream target genes to induce cell cycle arrest or cell death (Zilfou and Lowe, 2009). DNA damage induces the phosphorylation of p53 at several Ser/Thr-Pro residues, including Ser33, Ser46, Thr81 and Ser315 (Wulf et al., 2002; Zacchi et al., 2002; Zheng et al., 2002). Binding to Pin1 to phosphorylated p53 and the subsequent Pin1-mediated isomerization of p53 prevent the interaction of p53 with MDM2 since binding of Pin1 to pThr81-Pro motif of p53 disassociates p53 from MDM2, leading to stabilized p53 and the activation of p53 target genes (Zacchi et al., 2002).

The stability of p63 and p73, two other p53 gene family members, is also regulated by Pin1 (Mantovani et al., 2004; Li et al., 2013; Restelli et al., 2014). The conformation of p73 is altered by Pin1-mediated isomerization, promoting its interaction with p300 and the subsequent acetylation in a c-Abl dependent manner, likely preventing the ubiquitination of p73 on the acetylated lysine (Mantovani et al., 2004). As a result, Pin1 augments p73’s ability to induce the expression of proapoptotic genes, including *Bax*, *Pig3*, and *p53AIP1* (Mantovani et al., 2004). On the other hand, Pin1 specifically interacts with Thr538-Pro of p63a and disrupts the interaction between p63a and WWP1, an E3 ligase for p63a, resulting in the enhanced transcriptional activity for the expression of proapoptotic gene *Bax* (Li et al., 2013). It appears that Pin1 represents a common mediator linking proapoptotic cooperative activity of the p53 family members. As a regulator of p53, Pin1 regulates many cellular responses related to cell cycle and cell death, including genotoxic response, apoptosis, and mitochondrial apoptotic function (Wulf et al., 2002; Zacchi et al., 2002; Zheng et al., 2002; Follis et al., 2015; Mantovani et al., 2015).

Pin1 has been demonstrated to increase the stability of c-Jun via inhibition of its ubiquitination (Pulikkan et al., 2010). Pin1 binds to c-Jun that is phosphorylated on Ser63/73-Pro motifs by JNK or Ras (Wulf et al., 2001). Similar to p53, Pin1-mediated isomerization and the conformation change of c-Jun weakens its binding to the E3 ubiquitin ligase Fbw7, therefore attenuating the degradation of c-Jun (Csizmok et al., 2018). A similar mechanism is also identified for Pin1-mediated stabilization of estrogen receptor a(ERa), a key player in the development of breast cancer. ERa is phosphorylated at Ser118-Pro119 and Pin1 binds to this specific phosphorylated serine and induces the *cis-trans* isomerization of Pro119. Binding of Pin1 to ERa disrupts the ubiquitination of ERa by interfering with its interactions with the E3 ligase, E6AP, which is shown to bind to phosphorylated Ser118 and degrade ERa (Rajbhandari et al., 2014).

While the above examples confirm a role for Pin1 in the stabilization of transcription factors, Pin1 also promotes the degradation of transcription factors. Phosphorylation of Thr58 of c-Myc is critical for its oncogenic potential, since a mutation at Thr58 is often identified in the amplified *c-myc* genes in Burkitt’s lymphoma and Thr58 mutant of c-Myc demonstrates enhanced oncogenic potential with increased protein stability (Farrell and Sears, 2014). Phosphorylation of Thr58 is important for the recognition of c-Myc by Pin1 via the WW domain, which might lead to the conformational change of c-Myc, facilitating c-Myc dephosphorylation at Ser62 by PP2A and promoting c-Myc turnover by the ubiquitin-proteasome pathway (Yeh et al., 2004; Farrell et al., 2013). Therefore, Pin1 triggers the degradation of c-Myc by facilitating the dephosphorylation of c-Myc by PP2A. The increased protein stability and oncogenic potential of Thr58 mutant in Burkitt’s lymphoma might result from the defect in Pin1-medaited dephosphorylation of c-Myc.

Pin1 also reduces the stability of tumor suppressive transcription factors. RUNX3, a tumor suppressor in breast cancer (Chen, 2012), has been identified as a Pin1 substrate. Pin1 recognizes four phosphorylated Ser/Thr-Pro motifs in RUNX3 via its WW domain and reduces the cellular levels of RUNX3 in an isomerase activity-dependent manner by inducing the ubiquitination and proteasomal degradation of RUNX3 (Nicole Tsang et al., 2013). These four motifs are located immediately C-terminal of the runt domain, a region has been shown to be important for RUNX3 stability. Binding of Pin1 to these phosphorylated motifs and the associated conformational change of RUNX3 might result in the recruitment of RUNX3 E3 ligases (Nicole Tsang et al., 2013). Therefore, Pin1-mediated protein degradation might partially account for the decreased RUNX3 expression, an early event in breast cancer progression (Chuang and Ito, 2010). Interestingly, Pin1 also regulates the activity of RUNX2, which is another key member of the Runt family proteins and the master transcription factors for bone formation (Lian and Stein, 2003). Different from RUNX3, binding of Pin1 to phosphorylated RUNX2 stabilizes RUNX2 protein by preventing RUNX2 ubiquitination and degradation (Lee et al., 2013; Yoon et al., 2013). Through modulating the stability and transcriptional activity of RUNX2, Pin1 regulates the osteoblast differentiation and skeletal development (Lee et al., 2013; Yoon et al., 2013).

Other transcription factors regulated by Pin1 at the level of protein stability include RelA (Ryo et al., 2003), β-catenin (Ryo et al., 2001), IRF3 (Saitoh et al., 2006), Naong (Moretto-Zita et al., 2010), Oct4 (Nishi et al., 2011), MEF2C (Magli et al., 2010), SP1 (Yang et al., 2014), Osterix (Lee et al., 2015), ATF1 (Huang et al., 2016), TR3 (Chen et al., 2012), FoxM1 (Kruiswijk et al., 2016; Wang et al., 2016), Smad3 (Nakano et al., 2009), RAR (Gianni et al., 2009), FoxO3 (Shimizu et al., 2016), PPARγ (Fujimoto et al., 2010; Han Y. et al., 2016), and HIF-1a (Han H. J. et al., 2016) (Table 1). The detailed mechanisms for how Pin1 regulates their stability might be different for each factor, it appears that changing the accessibility of E3 ligases to the Pin1 substrates due to Pin1-medaited protein conformational change via isomerization might represent a general mechanism for the regulation of protein stability by Pin1. In this regard, Pin1 prevents the binding of E3 ligase RNF4 to SP1 and SPOP for Naong, respectively (Yang et al., 2014; Zhang et al., 2019). By changing the stability of these transcription factors and their transcriptional activities, Pin1 regulates diverse biological processes, including inflammatory response, cell proliferation, stem cell reprogramming, myogenesis, and bone formation (Table 1) (Liou et al., 2011).

### DNA Binding and Transactivation

DNA binding domain and transactivation domain (TAD) are two essential protein domains that help define a transcription factor. Pin1 is able to modulate both the DNA binding and transcriptional activity of transcription factors. Pin1 binds to the N-terminal Ser118-Pro motif in the intrinsic activation function 1 (AF1) domain of ERα (Rajbhandari et al., 2012). Binding of Pin1 and the subsequent Pin1-mediated conformational change via isomerization increases ERα DNA binding activity with a concomitant increase in ERα transcriptional activity in estrogen activated breast cancer cells (Rajbhandari et al., 2015). Pin1 also promotes the binding of c-Myc to the DNA, independent of the protein stability regulated by Pin1 (Farrell et al., 2013). This regulation requires Pin1 PPIase activity and the phosphorylation of c-Myc on Ser62-Pro63. While Pin1 stimulates the DNA binding activity of ERa and c-Myc, but the Pin1 binding motifs on ERa or c-Myc are not within the DNA binding domain (Farrell et al., 2013; Rajbhandari et al., 2015). How Pin1-mediated isomerization in one region could affect the activity of the DNA binding domain on a different region? One possibility is that the conformation change-mediated recruitment of co-activators (e.g., p300 and GCN5) might alter the accessibility of the chromatin, leading to the enhanced DNA binding of the transcription factors and the enhanced transcription of target genes. In this regard, the AF1 domain of ERa is responsible for the recruitment of SRC1 and CBP (Dutertre and Smith, 2003). c-Myc’s interaction with p300 and the recruitment of p300, GCN5, hSNF5, and pTEFb to promoters is also facilitated by the binding of Pin1 (Farrell et al., 2013; Sanchez-Arevalo Lobo et al., 2013). It has to be noted that Pin1 can also decrease the DNA binding activity of transcription factors. Binding of Pin1 to phosphorylated Thr739 of Sp1 has been reported to cause Sp1 to move out of the chromosome completely by decreasing its DNA binding activity during mitosis (Yang et al., 2014).

While changing the DNA binding affinity would affect the transcriptional activity of a transcription factor, Pin1 can also regulate the transcriptional activity by directly binding to the TADs of the transcription factors (Monje et al., 2005; Lufei et al., 2007; van Tiel et al., 2012; Lv et al., 2013; Poolman et al., 2013; Nakada et al., 2019) (Table 1). Phosphorylation of the carboxyl-terminal transactivation domain of c-Fos by extracellular signal-regulated kinases (ERK) in response to growth factors is essential for the transcriptional activation of AP-1, heterodimer of c-Jun and c-Fos (Monje et al., 2003). Pin1 binds to c-Fos through specific pSer/Thr-Pro sites within the c-Fos TAD, and this interaction results in an enhanced transcriptional response of c-Fos to polypeptide growth factors that stimulate ERK (Monje et al., 2005). The detailed mechanism for this enhanced transactivation is undetermined, but likely results from the change of interactions from transcription related proteins of the TAD (Monje et al., 2005).

Pin1 has also been shown to regulate the transcriptional activity of glucocorticoid receptor (GR) by binding to the TAD. Binding of Pin1 to the phosphorylated Ser203 and Ser221 within the TAD of GR enhances the transactivation of GR (Poolman et al., 2013). Interestingly, this enhanced transactivation appears to result from enhanced recruitment of GR to the promoters of its GR target genes but not directly from the transactivation (Poolman et al., 2013). How the binding of Pin1 to the TAD enhances the DNA binding activity of GR remains to be determined. It is possible that Pin1-mediated conformational change of TAD would affect the conformation of DNA binding domain, which is adjacent to the TAD (Poolman et al., 2013).

Pin1 also regulates the transcriptional activity of HIF-1a. Pin1 interacts with p42/p44 MAPK-mediated phosphorylation of HIF-1a at Ser641 and Ser643 of the transactivation region and promotes its conformational changes for the efficient expression of HIF-1a genes, including VEGF, GLUT1 and PGK1 (Jalouli et al., 2014). It has been speculated that the enhanced transactivation of HIF-1a might stem from the increased HIF-1a binding to DNA or transcriptional cofactors (Jalouli et al., 2014).

### Pin1 and the Transcription Co-regulators

Transcription factors often recruit transcription co-activators for their full transcriptional potential and biological functions (Spiegelman and Heinrich, 2004). Pin1 regulates the activity of some transcription co-regulators to control the effective gene expression.

Steroid receptor-mediated transcription requires the ligand-dependent association of receptors with steroid receptor coactivator 3 (SRC-3) (Lydon and O’malley, 2011). Pin1 interacts with phosphorylated SRC-3 and regulates its co-activation function by enhancing its interaction with CBP/p300 and stimulating its cellular turnover, facilitating the cyclic recruitment of nascent phosphorylated SRC-3 to the promoter (Yi et al., 2005).

Pin1 also regulates CREB co-activator CRTC2 (CREB-regulated transcriptional co-activator 2) by binding to phosphorylated CRTC2 at Ser136, which locates within its nuclear localization signal (Nakatsu et al., 2010). Different from SRC-3, binding of Pin1 to phosphorylated CRTC2 suppresses the co-activation function of CRTC2 by attenuating its nuclear localization and cAMP-responsive element (CRE) transcriptional activity (Nakatsu et al., 2010).

A recent study also demonstrates that transcriptional co-activator PRDM16 is negatively regulated by Pin1 (Nakatsu et al., 2019). PRDM16 plays crucial roles in the determination and function of brown and beige fat as well as in hematopoiesis and cardiac development (Chi and Cohen, 2016). Pin1 interacts with phosphorylated PRDM16 at Ser44A, Ser52A, Thr61A and Ser66A, promotes its degradation and the suppression of the thermogenic response (Chi and Cohen, 2016). The detailed mechanism for Pin1-mediated PRDM16 degradation remains undetermined. Nevertheless, by regulating the activity of co-activators such as CRTC2 and PRDM16, Pin1 is involved in the regulatory mechanism governing the glucose metabolism and adipose thermogenesis (Nakatsu et al., 2016).

In the Notch1 signaling pathway, activation of CSL [CBF-1, Su(H), Lag-1] -target genes requires the co-activation function of the intracellular domain of the notch protein (NICD), which is released from the membrane-bound Notch1 protein processed by the γ-secretase (Bray, 2016). NICD has also been shown to be a co-activator for Notch-mediated activation of LEF-1 target gene independent of its co-activation function for CSL (Ross and Kadesch, 2001). Binding of Pin1 to Notch1 stimulates the processing of the Notch1 from its inactive transmembrane form to γ-secretase-processed, activated nuclear localized form (Rustighi et al., 2009). The catalytic activity of Pin1 is required for the cleavage of the Notch protein by γ-secretase for the release of NICD (Rustighi et al., 2009). By mediating the generation of NICD, Pin1 regulates gene expression in Notch signaling pathway.

In addition to the regulation of transcription co-activators, Pin1 can control gene expression by targeting transcription co-repressors. Silencing mediator for retinoic acid and thyroid hormone receptor (SMRT) is a transcriptional corepressor that participates in diverse signaling pathways and human diseases (Chen and Evans, 1995). Pin1 interacts with SMRT and regulates SMRT protein stability, thereby affecting SMRT-dependent transcriptional repression (Stanya et al., 2008). SMRT is phosphorylated by Cdk2 at Ser1241, Ser1445 and Ser1469. Cdk2-mediated phosphorylation of SMRT at these serines is required for Pin1 binding and the decreased SMRT stability. More importantly, ErbB2 destabilizes SMRT protein level via Cdk2-Pin1 axis, suggesting that ErbB2 signaling upstream of Cdk2 and Pin1 is a potential regulatory cascade involved in regulating the stability of SMRT (Stanya et al., 2008). Interestingly, two of the Cdk2 phosphorylation sites of the Pin1 binding motifs in SMRT are conserved in N-CoR, a closely related transcription repressor (Stanya and Kao, 2009), suggesting that the activity of N-CoR might be regulated by Pin1 via a similar mechanism.

## Pin1 and RNA Polymerase II

Transcription factors and transcription coactivators are essential for the recruitment of RNAPII to the promoters or enhancers to activate transcription. The C-terminal domain (CTD) of Rpb1, the largest subunit of RNAPII, which consists of 26-52 tandem heptapeptide repeats with the general consensus sequence Tyr_1_-Ser_2_-Pro_3_-Thr_4_-Ser_5_-Pro_6_-Ser_7_ from yeast to human. The proline-rich CTD functions as a docking platform for numerous transcription regulatory proteins involved in transcription initiation, elongation, termination and post-transcription processing (Hahn, 2004). The CTD is marked by a number of post-translational modifications, including phosphorylation, glycosylation, methylation, and acetylation (Brookes and Pombo, 2009). During the early events of transcription initiation, unphosphorylated RNAPII, general transcription factors and a mediator complex are recruited onto the promoters to form the pre-initiation complex (PIC) (Thomas and Chiang, 2006). Phosphorylation of Ser5 promotes the dissociation of RNAPII from PIC and the promoter clearance, processes that are required for transition from initiation to early elongation (Phatnani and Greenleaf, 2006; Sogaard and Svejstrup, 2007). Different from phosphorylated Ser5, phosphorylation of Ser2 results in the recruitment of elongation, termination and 3′ end processing factors, allowing the coupling of transcription elongation with mRNA processing (Bentley, 2002; Ahn et al., 2004).

Pin1 binds to both pSer2-Pro3 and pSer5-Pro6 of the CTD (Xu et al., 2003; Zhang et al., 2012). Pin1’s binding to CTD depends on the phosphorylation of Ser2 by kinases CDK2 and CDK9 (Komarnitsky et al., 2000; Bartkowiak et al., 2010), and the phosphorylation of Ser5 by CDK7 (Phatnani and Greenleaf, 2006). Pin1 induces the conformational changes of the CTD, leading to the recruitment of CTD-modifying enzymes and transcription regulatory proteins essential for RNAPII function (Hanes, 2015). The presence of two Ser-Pro motifs with the CTD repeats creates four possible *cis*-*trans* configurations, and thus expands the complexity of the CTD code signature by providing a scaffold for the recruitment of a variety of chromatin and RNA processing factors (Srivastava and Ahn, 2015). The *cis* or *trans* configuration of the Pin1 binding motifs on CTD determines the transcription outcome via the recruited factors. For example, Mce (capping enzyme), Pcf11 (3′ end processing factor), Scp1 (CTD phosphatases) and SCAF8 (splicing factor) bind to phosphorylated CTD with the prolines in *trans* configuration (Fabrega et al., 2003; Noble et al., 2005; Zhang et al., 2006; Becker et al., 2008; Ghosh et al., 2011). In contrast, Ssu72 and the termination factor Nrd1 bind CTD with phosphorylated Ser5-pro6 in the *cis* configuration (Xiang et al., 2010; Werner-Allen et al., 2011; Kubicek et al., 2012).

RNAPII is subject to regulatory control at all steps of transcription cycle, including initiation, elongation and termination. The high selectivity of transcription regulatory proteins for *cis* or *trans* isomers supports the idea that Pin1 serves as a key transcription regulator for gene expression. However, how Pin1 creates and maintains the *cis* or *trans* configuration of CTD in during transcription cycle remains obscure and merit further investigation.

## Pin1 and Transcription Initiation

Transcription initiation encompasses multiple steps, including the exposure of promoters in chromatin, the association of promoters with RNAPII and transcription regulatory proteins to form PIC, and the clearance of promoter for the release of RNAPII (Li et al., 2007). The wrapping of promoter DNA around a histone octamer in the nucleosome suppresses transcription initiation. The ordered disassembly of nucleosomes facilitates transcription by allowing RNAPII to interact with the promoters. Histone H1 plays a crucial role in maintaining higher order chromatin structure and reversible phosphorylation of H1 is closely correlated with transcription initiation with increased phosphorylation of H1 associating with a relaxed chromatin structure, allowing the access of RNAPII and DNA-binding proteins to the promoter region (Hohmann, 1983; Vicent et al., 2011).

Pin1 has been demonstrated to bind to H1 via phosphorylated S/T-Pro residues on the C-terminal of H1 (Raghuram et al., 2013). Pin1 promotes dephosphorylation of H1 and stabilizes H1’s interaction with chromatin to facilitate condensation, implying that Pin1 may act as a suppressor of transcription initiation. The idea that Pin1 inhibits transcription initiation is supported by *in vitro* transcription assays demonstrating that Pin1 inhibits transcription initiation in nuclear extracts whereas an inactive Pin1 mutant stimulates transcription initiation (Xu and Manley, 2007a). Pin1 might also inhibit transcription initiation via dephosphorylation of Ser5 of the CTD of RNAPII (Werner-Allen et al., 2011). However, some studies indicate that Pin1 might have a positive effect on transcription initiation since Pin1 inhibitor Juglone disrupts the formation of functional PIC (Chao et al., 2001). The discrepancy in these studies might result from the different *in vitro* and *in vivo* assays and the approaches to inhibit Pin1. For example, Pin1 inhibitor Juglone is known to be toxic to the cells and might have off-target effects, which accounts for the initiation inhibition (Nakatsu et al., 2018). Pin1 has also been shown to regulate the chromosome condensation during mitosis targeting the topoisomerase (Topo) IIa (Xu and Manley, 2007a).

While Pin1 indirectly regulates the transcription initiation by affecting the chromosome structure, it is also possible that Pin1 might directly affect the activity of transcription initiation factors. The activity of transcription initiation factor TFIID is tightly regulated by phosphorylation. During mitosis, TFIID is phosphorylated at multiple sites and phosphorylated TFIID is unable to direct activator-dependent transcription (Segil et al., 1996). Considering that Pin1 is a major regulator of mitosis (Liou et al., 2011), Pin1 might target TFIID to regulate transcription during cell cycle. Interestingly, mice deficient in TAF4b, a gonad-specific subunit of TAFIID exhibit germ cell deficiency, a phenotype similar to Pin1^–/–^ mice (Falender et al., 2005). These studies provide genetic evidence linking Pin1 to TFIID, but the detailed mechanism how Pin1 regulates TFIID for the transcription initiation needs to be further investigated.

## Pin1 and Transcription Elongation

After ∼20–60 bp RNA is synthesized, RNAPII is repressed by negative elongation factors, such as DSIF (DRB sensitivity-inducing factor) and NELF (negative elongation factor) at promoter-proximal pausing sites (Wada et al., 1998; Yamaguchi et al., 1999). CDK9, a catalytic subunit of P-TEFb (positive transcriptional elongation factor b), is recruited and activated by Brd4 (Bromodomain-containing protein 4), and phosphorylates NELF, DSIF and Ser2 in the CTD of RNAPII (Li Y. et al., 2018). Phosphorylation of NELF and DSIF by CDK9 removes these negative factors from the pausing sites, releasing the paused RNAPII into the productive elongation phase (Fujinaga et al., 2004). Pin1 seems to play a role in the transcription elongation by removing the negative elongation factor DSIF and activating the positive elongation factor P-TEFb.

Pin1 binds to the hSpt5 subunit of DSIF via its phosphorylated carboxyl terminal part 2 (CTR2) domain by Cdk9 (Lavoie et al., 2001). The CTR2 domain contains a p(T/S)PSP(Q/A)(S/G)Y motif, which resembles the CTD repeats of RNAPII (Hanes, 2015). hSpt5 is phosphorylated by CDK9 in interphase but not in mitosis and this interphase form of phosphorylated hSpt5 is bound to the nuclear matrix, indicating its involvement in transcription (Lavoie et al., 2001). Binding of Pin1 to phosphorylated hSpt5 induces the conformational change of hSpt5 via isomerization, leading to the subsequent change of its phosphorylation status and the conversion of DSIF from a repressor to an activator (Lavoie et al., 2001).

In mammalian cells, Brd4 regulates transcription elongation by recruiting P-TEFb to stimulate the phosphorylation of the CTD of RNAPII (Jang et al., 2005; Ai et al., 2011). Brd4 has emerged as an important factor in tumorigenesis by promoting the transcription of genes involved in cancer development (Muller et al., 2011; Wu et al., 2013; Basheer and Huntly, 2015; Jung et al., 2015). Our recent studies demonstrate that the stability and functions of Brd4 are positively regulated by Pin1 in cancer cells (Hu et al., 2017). Pin1 directly binds to phosphorylated Thr204 of Brd4 by an unidentified kinase and enhances Brd4’s stability by inhibiting its ubiquitination. Pin1 also catalyzes the isomerization of Pro205 of Brd4 and induces its conformational change through a *cis-trans* isomerization, which leads to enhanced CDK9 binding to Brd4 and enhanced recruitment of CDK9 to a subset of promoters of Brd4-mediated tumor-promoting genes, including c*-MET* and *MMP9* (Hu et al., 2017). In addition to the enhanced CDK9 binding, Pin1-mediated conformational change might also decrease the accessibility of a Brd4 E3 ligase or increase the accessibility of a Brd4 deubiquitinating enzyme for the increased protein stability with reduced ubiquitination (Hu et al., 2017). Therefore, the overall tumor-promoting activity of Brd4 in cancer cells might result from the Pin1-mediated conformational change of Brd4, leading to more stabilized Brd4 and conformational change-associated transcriptional potential increase (Hu et al., 2017).

## Pin1 and Transcription Termination

Termination of transcription involves the release of RNA transcripts, the dissociation of RNAPII and its binding proteins from the DNA, coupled with the cleavage of 3′ end of the nascent transcript and the polyadenylation (Richard and Manley, 2009). Phosphorylation of RNAPII at Ser2 or Ser5 is closely related to transcription termination. Levels of Ser5 phosphorylation is high near the transcription start site, while Ser2 phosphorylation increases over the gene body, peaking near the transcription termination site (Hsin and Manley, 2012). Pin1 increases the dephosphorylation of Ser5, but not Ser2, by CTD phosphatase Ssu72 (Kops et al., 2002; Werner-Allen et al., 2011). Pin1 also inhibits the CTD dephosphorylation by affecting the activity of another CTD phosphatase, Fcp1, or increasing the CTD phosphorylation by Cdc2/Cyclin B (Xu and Manley, 2007a, b). As such, Pin1 can regulate the transcription termination by changing the phosphorylation status of CTD of RNAPII.

Various phosphorylation status of CTD of RNAPII creates a CTD code that dictates the assembly and disassembly of factors to the RNAPII and determines the transcription outcome. Pin1 has been implicated in the construction and deciphering the CTD code (Buratowski, 2003). In yeast, mRNA 3′-end processing factor Pcf11 binds to CTD repeats of RNAPII containing Pro3 in the *trans*-configuration (Noble et al., 2005), whereas the termination factor Nrd1 binds to the *cis* form of phosphorylated Ser5 (Kubicek et al., 2012). By changing the *cis-* or *trans*-configuration of prolines in CTD and coordinating the recruitment of the termination and/or 3′ -end mRNA processing factors, such as Pcf11, Rtt103 and Nrd1 (Noble et al., 2005; Lunde et al., 2010; Kubicek et al., 2012), Ess1 (yeast Pin1) might facilitate the transcription termination. A similar regulator mechanism might also occur in mammalian cells since these CTD binding factors are highly conserved in eukaryotic cells.

## Pin1 and the Post-Transcriptional Regulation of Gene Expression

mRNA levels are determined by a complex interplay between the rates of gene transcription and mRNA decay (Schoenberg and Maquat, 2012). mRNA decay is closely associated with the 3′ untranslated region (3′-UTR) of the mRNAs. Many early response genes contain AU-rich element (ARE) in 3′-UTRs (Barreau et al., 2005). AREs occur in up to 5–8% of all mRNA transcripts in human cells and these AREs are recognized by AU-binding proteins (AUBPs), which promote either decay or stabilization of mRNA on a gene- and cell type-specific manner (Barreau et al., 2005; Halees et al., 2008). Many AUBPs are phosphoproteins and their activity is tightly regulated through reversible phosphorylation (Shen and Malter, 2015). Via binding to the specific phosphorylated AUBPs, Pin1 controls mRNA decay of selective genes.

Histone mRNAs are rapidly degraded at the end of S phase, and a 26-nucleotide stem-loop in the 3′-UTR is a key determinant of histone mRNA stability (Heintz et al., 1983). This sequence is the binding site for stem-loop binding protein (SLBP), which helps to recruit components of the RNA degradation machinery to the histone mRNA (Wang et al., 1996). Pin1 binds to phosphorylated Thr171-Pro172 of SLBP and promotes its dephosphorylation by PP2A, causing its dissociation from histone mRNA hairpin, triggering the rapid degradation of histone mRNA (Krishnan et al., 2012). Another example for Pin1-mediated mRNA stability is the mRNA of the parathyroid hormone (PTH), which regulates the serum calcium via its effect on bone, kidney, and intestine (Nechama et al., 2009). The stability of *PTH* mRNA is decreased by the binding of K-homology splicing regulator protein (KSRP) to a *cis*-acting element in the 3′-UTR region of *PTH* mRNA (Nechama et al., 2008). Pin1 interacts with the phosphorylated Ser181 of KSRP and induces the *cis-trans* isomerization of the proline bond in KSRP. The conformational change of KSRP exposes the phosphorylated Ser181, triggering the dephosphorylation, an event that is required for the activation of KSRP. Activated KSRP then interacts with *PTH* mRNA and induces its decay (Nechama et al., 2009).

Pin1 can also regulate the mRNA stability of cytokine via binding to AUBPs. AUF1 typically functions as a destabilizing protein for AU-rich mRNAs, including *GM-CSF* and *c-Fos* (Loflin et al., 1999). Pin1 associates with phosphorylated AUF1 and disassociates AUF1 from the mRNA of *GM-CSF* in activated eosinophils and T cells (Shen et al., 2005; Esnault et al., 2006). Binding of Pin1 to AUF1 changes the conformation of AUF1 and attenuates its RNA binding activity, leading to the stabilization of *GM-CSF* mRNA by HuR or hnRNP C (Shen et al., 2005; Esnault et al., 2006). Via a similar mechanism, Pin1 regulates the stability of *TGF-β1* mRNA and *c-Fos* mRNA (Shen et al., 2008; Krishnan et al., 2014). Regulation of mRNA stability by targeting specific RNA binding proteins, including AUF1, KSRP, SLBP, and HuR, might represent another layer of gene regulation by Pin in cancer and inflammatory response.

MicroRNAs (miRNAs) are small, endogenous non-coding RNAs of 18–24 nucleotides in length and play significant roles in the regulation of gene expression and participate in numerous cellular processes, including cell cycle arrest, cell proliferation and death (Benhamed et al., 2012). miRNAs bind to the 3′-UTR of target mRNAs via nucleotide pairing and regulates the target gene expression by decreasing the mRNA stability or translation (Fabian et al., 2010). The biogenesis of miRNAs is tightly controlled at multiple steps, including RNAPII-dependent transcription of miRNA genes, Drosha- and Dicer-mediated processing of primary miRNAs (pri-miRNAs) or precursor miRNAs (pre-miRNA), and the nuclear export of (pre-miRNAs) to the cytoplasm by exportin-5 (XPO5) (Ha and Kim, 2014). Recent studies demonstrate that the biogenesis of miRNAs, especially the XPO5-mediated export of pre-miRNA, is regulated by Pin1 (Li J. et al., 2018; Pu et al., 2018). Pin1 binds to the ERK-mediated phosphorylated XPO5 in hepatocellular carcinoma (HCC) and changes XPO5’s conformation through *cis-trans* isomerization, leading to the retention of XPO5 in the nucleus and the impaired nuclear export of pre-miRNAs (Li J. et al., 2018). As a result, several tumor suppressor miRNAs, including miR-200b, miR-146a and miR-122, are down-regulated in HCC (Li J. et al., 2018). Down-regulation of these miRNAs likely changes the expression of their target genes, promoting the development of HCC (Li J. et al., 2018; Pu et al., 2018). Therefore, Pin1 is also able to regulate gene expression at the post-transcriptional level via controlling the biogenesis of miRNAs, adding another regulatory layer for mRNA stability.

## Summary and Perspectives

**FIGURE 1:**
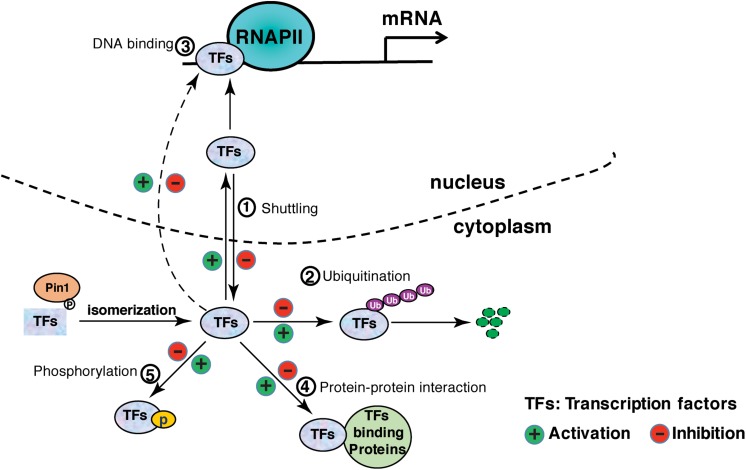
Pin1 regulates the activation of transcription factors via distinct mechanisms: ① affects nucleocytoplasmic shuttling; ② affects protein stability by ubiquitination; ③ affects DNA-binding affinity; ④ affects protein interaction; ⑤ regulates phosphorylation or dephosphorylation.

**FIGURE 2:**
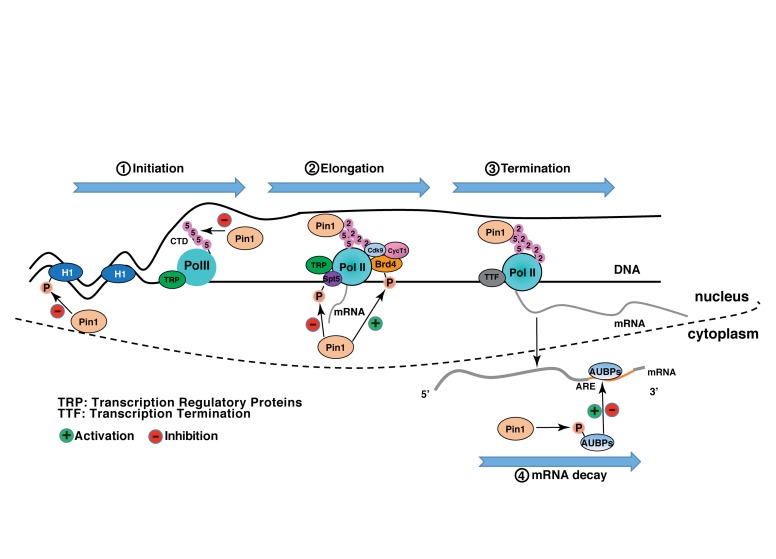
Pin1 regulates gene expression network. During transcription initiation, Pin1 promotes dephosphorylation of Histone H1 and Ser5 in the CTD of RNA polymerase II, to inhibit the recruitment of TRP (transcription regulatory proteins) and pre-initiation complex, and promoter clearance, processes that are required to transcription initiation. During transcription elongation, on one hand, Pin1 binds to phosphorylated Spt5 and might facilitate conversion of DSIF from a negative elongation factor into a positive elongation factor. On the other hand, Pin1 enhances Brd4’s stability and its interacting with CDK9 to phosphorylate Ser2 in the CTD of RNA polymerase II, and thus increases transcription elongation. In transcription termination, Pin1 binds to phosphorylated Ser2 in the CTD of RNAPII and facilitates coordinate recruitment of TTF (transcription termination factors). After the synthesis of mRNA, Pin1 binds to AUBPs (AU-binding proteins) to accelerate or slow mRNA decay.

Pin1 is involved in almost every step of gene expression, from activation of transcription factors to transcription initiation and termination by targeting a host of transcription factors and transcription regulatory proteins (Table 1). In many cases, Pin1 binds to the phosphorylated transcription factors and induces the protein conformational change via isomerization, although direct evidence for the isomerization-mediated conformational change is missing in some of the studies (Table 1). Conformation change accounts for the changes of various protein properties, including protein stability, subcellular localization, phosphorylation status, protein-protein interactions, protein-DNA interactions, leading to the increased or decreased transcriptional potential of these transcription factors (Figure 1). In addition, Pin1 also targets RNA polymerase II through interacting with the CTD of Rbp1. Pin1-mediated isomerization of prolines or phosphorylation status of CTD generates a CTD code for recruitment or disengagement of transcription regulatory proteins required for transcription initiation, elongation and termination (Figure 2). Finally, Pin1 controls mRNA decay by interacting with AUBPs (Figure 2). It has to be noted that Pin1 often affects the activity of a single substrate via multiple mechanisms. For example, Pin1 regulates the nucleocytoplasmic shuttling and the stability of RelA and β-catenin (Ryo et al., 2001, 2003). Pin1 also regulates both the stability and DNA binding activity ERa (Rajbhandari et al., 2014, 2015). Via these multi-level regulations, Pin1 might impose the spatiotemporal control of the expression of a subset of genes.

Epigenetics plays crucial roles in the regulation of gene expression by post-translational modifications of histone proteins and methylation of DNA (Dawson et al., 2012). Epigenetic regulation is mediated by various enzymes that add or remove various modifications (writers and erasers) and the proteins that recognize these modifications (readers) (Dawson et al., 2012). While some PPIases regulating histone modifying enzymes have been reported (Hanes, 2015), studies on epigenetic regulation of gene expression by Pin1 are largely missing. We have recently shown that epigenetic reader Brd4, which specifically binds to the acetylated lysine on histone and non-histone proteins, is a Pin1 substrate and the stability and transcriptional activity of Brd4 is regulated by Pin1-catalyzed isomerization (Hu et al., 2017). Whether and how Pin1 regulates gene expression via targeting these epigenetic regulators remain exciting questions and need to be further investigated. Many of these epigenetic factors are dysregulated in cancer and the highly expressed Pin1 in cancer might contribute to the dysregulation.

Pin1-catalyzed isomerization and the subsequent protein conformational change might accounts for all the functional changes of Pin1 substrates. Protein conformational change often leads to the engagement or disassociation of the interacting proteins. The alteration of protein stability after conformational change is largely affected by the changes in the accessibility to E3 ligases. Conformational change can also alter the accessibility of the NLS or NES to the nuclear import or export machinery, affecting the nucleocytoplasmic shuttling of the transcription factors. However, how Pin1 directly regulates the DNA binding activity via protein conformational change is not quite clear. Although it is generally believed that binding of Pin1 leads to the conformational changes of its substrates, many studies failed to include the isomerase inactive mutant of Pin1 (Table 1), an issues needs to be addressed in the future studies.

Pin1 could have completely opposite effects on its substrates. Pin1 increases or decreases the stability of transcription factors in a similar phosphorylation and isomerization-dependent manner (Table 1). Pin1 regulates the activities of a spectrum of transcription factors, many of which are oncogenes and tumor suppressors (Lu and Zhou, 2007). Pin1 is aberrantly activated in most cancers and Pin1 generally activates the oncogenic transcription factors but inhibits the tumor suppressive transcription factors, reflecting Pin1’s ability to promote cancer cells by activating caner promoting factors and inactivating cancer suppressive factors (Zhou and Lu, 2016). However, it is not clear how Pin1 imposes the opposite regulatory effects on oncogenic and tumor suppressive transcription factors. One possibility is that the expression of the target genes of these transcription factors and the resulting cellular functions might provide some feedback signals for Pin1 to determine the fates of these transcription factors.

While Pin1 is able to regulate gene expression at various levels, it is possible that Pin1 is not absolutely required for the transcription of whole genome. The success rate of Pin1^–/–^ homozygous cross breeding was much lower that that of heterozygous mice, indicating a critical role of Pin1 in gene expression and cell division (Liou et al., 2002). Consistently, Pin1^–/–^ fibroblasts grow normally but with defect in re-entering the cell cycle from G_0_ arrest (Fujimori et al., 1999; Liou et al., 2002). Pin1-mediated transcription and gene expression is clearly a cell type-specific and signal-dependent event since many transcription factors and their target genes are inducible in response to specific stimuli. Studies on the transcription regulation by Pin1 were largely performed *in vitro* or in cultured cells with recombinant or overexpressed Pin1. The significance of these biochemical studies in gene regulation would be strengthened if similar regulatory mechanism would be confirmed in Pin1^–/–^ or Pin1 conditional knockout mice in combination with mouse disease models. A great example is demonstrated in a recent study of the identification of Pin1 as a regulator of thermogenesis by targeting PRDM16 for degradation (Nakatsu et al., 2019).

While a great deal is known about how Pin1 regulates the activation transcription factors for gene expression in response to stimuli, much less is known about how Pin1 regulates the transcription machinery for the spatiotemporal control of gene expression except that Pin1 helps to construct the CTD code. It also remains to be determined whether these regulations on RNAPII and the associated transcription is a general mechanism that can apply to all genes or whether it is only a gene-specific and cell-specific phenomenon. Furthermore, the subcellular localization, the expression levels and the activity of Pin1 are subject to change in response to stimulation and in diseases conditions (Boussetta et al., 2010; Lee et al., 2011; Rangasamy et al., 2012; Zannini et al., 2019), adding another layer of complexity to Pin1-mediated gene expression. Overall, better understating the regulation of gene expression by Pin1 would provide new insights into the pathophysiological functions of Pin1 and new therapeutic approaches for the treatment of cancer and other human diseases by targeting Pin1 alone or in combination with targeting different transcription regulators.

## Author Contributions

XH drafted the manuscript. L-FC revised the manuscript. XH and L-FC reviewed and modified the manuscript. All authors agreed on the final version.

## Conflict of Interest

The authors declare that the research was conducted in the absence of any commercial or financial relationships that could be construed as a potential conflict of interest.

## References

[B1] Ahn S. H. Kim M. Buratowski S. (2004). Phosphorylation of serine 2 within the RNA polymerase II C-terminal domain couples transcription and 3′ end processing. *Mol. Cell* 13 67–76. 10.1016/s1097-2765(03)00492-1 14731395

[B2] Ai N. Hu X. Ding F. Yu B. Wang H. Lu X. (2011). Signal-induced Brd4 release from chromatin is essential for its role transition from chromatin targeting to transcriptional regulation. *Nucleic Acids Res.* 39 9592–9604. 10.1093/nar/gkr698 21890894 PMC3239188

[B3] Atkinson G. P. Nozell S. E. Harrison D. K. Stonecypher M. S. Chen D. Benveniste E. N. (2009). The prolyl isomerase Pin1 regulates the NF-kappaB signaling pathway and interleukin-8 expression in glioblastoma. *Oncogene* 28 3735–3745. 10.1038/onc.2009.232 19668231 PMC5987556

[B4] Barreau C. Paillard L. Osborne H. B. (2005). AU-rich elements and associated factors: are there unifying principles? *Nucleic Acids Res.* 33 7138–7150. 10.1093/nar/gki1012 16391004 PMC1325018

[B5] Bartkowiak B. Liu P. Phatnani H. P. Fuda N. J. Cooper J. J. Price D. H. (2010). CDK12 is a transcription elongation-associated CTD kinase, the metazoan ortholog of yeast Ctk1. *Genes Dev.* 24 2303–2316. 10.1101/gad.1968210 20952539 PMC2956209

[B6] Basheer F. Huntly B. J. (2015). BET bromodomain inhibitors in leukemia. *Exp. Hematol.* 43 718–731. 10.1016/j.exphem.2015.06.004 26163798

[B7] Becker R. Loll B. Meinhart A. (2008). Snapshots of the RNA processing factor SCAF8 bound to different phosphorylated forms of the carboxyl-terminal domain of RNA polymerase II. *J. Biol. Chem.* 283 22659–22669. 10.1074/jbc.m803540200 18550522

[B8] Benhamed M. Herbig U. Ye T. Dejean A. Bischof O. (2012). Senescence is an endogenous trigger for microRNA-directed transcriptional gene silencing in human cells. *Nat. Cell Biol.* 14 266–275. 10.1038/ncb2443 22366686 PMC5423543

[B9] Bentley D. (2002). The mRNA assembly line: transcription and processing machines in the same factory. *Curr. Opin. Cell Biol.* 14 336–342. 10.1016/s0955-0674(02)00333-2 12067656

[B10] Boussetta T. Gougerot-Pocidalo M. A. Hayem G. Ciappelloni S. Raad H. Arabi Derkawi R. (2010). The prolyl isomerase Pin1 acts as a novel molecular switch for TNF-alpha-induced priming of the NADPH oxidase in human neutrophils. *Blood* 116 5795–5802. 10.1182/blood-2010-03-273094 20956805 PMC3031377

[B11] Bray S. J. (2016). Notch signalling in context. *Nat. Rev. Mol. Cell Biol.* 17 722–735. 10.1038/nrm.2016.94 27507209

[B12] Brenkman A. B. De Keizer P. L. Van Den Broek N. J. Van Der Groep P. Van Diest P. J. Van Der Horst A. (2008). The peptidyl-isomerase Pin1 regulates p27kip1 expression through inhibition of Forkhead box O tumor suppressors. *Cancer Res.* 68 7597–7605. 10.1158/0008-5472.can-08-1059 18794148

[B13] Brookes E. Pombo A. (2009). Modifications of RNA polymerase II are pivotal in regulating gene expression states. *EMBO Rep* 10 1213–1219. 10.1038/embor.2009.221 19834511 PMC2775184

[B14] Buratowski S. (2003). The CTD code. *Nat. Struct. Biol.* 10 679–680. 10.1038/nsb0903-679 12942140

[B15] Cartwright P. Helin K. (2000). Nucleocytoplasmic shuttling of transcription factors. *Cell Mol. Life Sci.* 57 1193–1206. 10.1007/pl00000759 11028912 PMC11146813

[B16] Chao S. H. Greenleaf A. L. Price D. H. (2001). Juglone, an inhibitor of the peptidyl-prolyl isomerase Pin1, also directly blocks transcription. *Nucleic Acids Res.* 29 767–773. 10.1093/nar/29.3.767 11160900 PMC30403

[B17] Chen H. Z. Li L. Wang W. J. Du X. D. Wen Q. He J. P. (2012). Prolyl isomerase Pin1 stabilizes and activates orphan nuclear receptor TR3 to promote mitogenesis. *Oncogene* 31 2876–2887. 10.1038/onc.2011.463 22002310

[B18] Chen J. D. Evans R. M. (1995). A transcriptional co-repressor that interacts with nuclear hormone receptors. *Nature* 377 454–457. 10.1038/377454a0 7566127

[B19] Chen L. F. (2012). Tumor suppressor function of RUNX3 in breast cancer. *J Cell Biochem.* 113 140–147. 10.1002/jcb.24074 PMC3337355 22275124

[B20] Chen L. F. Greene W. C. (2004). Shaping the nuclear action of NF-kB. *Nat. Rev. Mol. Cell Biol.* 5 392–401. 10.1038/nrm1368 15122352

[B21] Chi J. Cohen P. (2016). The multifaceted roles of PRDM16: adipose biology and beyond. *Trends Endocrinol. Metab* 27 11–23. 10.1016/j.tem.2015.11.005 26688472

[B22] Chuang L. S. Ito Y. (2010). RUNX3 is multifunctional in carcinogenesis of multiple solid tumors. *Oncogene* 29 2605–2615. 10.1038/onc.2010.88 20348954

[B23] Csizmok V. Montecchio M. Lin H. Tyers M. Sunnerhagen M. Forman-Kay J. D. (2018). Multivalent interactions with Fbw7 and Pin1 facilitate recognition of c-Jun by the SCF(Fbw7) Ubiquitin Ligase. *Structure* 26:e22. 10.1016/j.str.2017.11.003 29225075

[B24] Dawson M. A. Kouzarides T. Huntly B. J. (2012). Targeting epigenetic readers in cancer. *N Engl. J. Med.* 367 647–657. 10.1056/nejmra1112635 22894577

[B25] Dilworth D. Gudavicius G. Leung A. Nelson C. J. (2012). The roles of peptidyl-proline isomerases in gene regulation. *Biochem. Cell Biol.* 90 55–69. 10.1139/o11-045 21999350

[B26] Dong X. Greven M. C. Kundaje A. Djebali S. Brown J. B. Cheng C. (2012). Modeling gene expression using chromatin features in various cellular contexts. *Genome Biol.* 13:R53. 10.1186/gb-2012-13-9-r53 PMC3491397 22950368

[B27] Dutertre M. Smith C. L. (2003). Ligand-independent interactions of p160/steroid receptor coactivators and CREB-binding protein (CBP) with estrogen receptor-alpha: regulation by phosphorylation sites in the A/B region depends on other receptor domains. *Mol. Endocrinol.* 17 1296–1314. 10.1210/me.2001-0316 12714702

[B28] Esnault S. Shen Z. J. Whitesel E. Malter J. S. (2006). The peptidyl-prolyl isomerase Pin1 regulates granulocyte-macrophage colony-stimulating factor mRNA stability in T lymphocytes. *J. Immunol.* 177 6999–7006. 10.4049/jimmunol.177.10.6999 17082615

[B29] Fabian M. R. Sonenberg N. Filipowicz W. (2010). Regulation of mRNA translation and stability by microRNAs. *Annu. Rev. Biochem.* 79 351–379. 10.1146/annurev-biochem-060308-103103 20533884

[B30] Fabrega C. Shen V. Shuman S. Lima C. D. (2003). Structure of an mRNA capping enzyme bound to the phosphorylated carboxy-terminal domain of RNA polymerase II. *Mol. Cell* 11 1549–1561. 10.1016/s1097-2765(03)00187-4 12820968

[B31] Falender A. E. Freiman R. N. Geles K. G. Lo K. C. Hwang K. Lamb D. J. (2005). Maintenance of spermatogenesis requires TAF4b, a gonad-specific subunit of TFIID. *Genes Dev.* 19 794–803. 10.1101/gad.1290105 15774719 PMC1074317

[B32] Fan G. Fan Y. Gupta N. Matsuura I. Liu F. Zhou X. Z. (2009). Peptidyl-prolyl isomerase Pin1 markedly enhances the oncogenic activity of the rel proteins in the nuclear factor-kappaB family. *Cancer Res.* 69 4589–4597. 10.1158/0008-5472.can-08-4117 19458071 PMC2741415

[B33] Farrell A. S. Pelz C. Wang X. Daniel C. J. Wang Z. Su Y. (2013). Pin1 regulates the dynamics of c-Myc DNA binding to facilitate target gene regulation and oncogenesis. *Mol. Cell. Biol.* 33 2930–2949. 10.1128/mcb.01455-12 23716601 PMC3719674

[B34] Farrell A. S. Sears R. C. (2014). MYC degradation. *Cold Spring Harb. Perspect. Med.* 4:a014365. 10.1101/cshperspect.a014365 24591536 PMC3935390

[B35] Follis A. V. Llambi F. Merritt P. Chipuk J. E. Green D. R. Kriwacki R. W. (2015). Pin1-induced proline isomerization in cytosolic p53 mediates BAX activation and apoptosis. *Mol. Cell* 59 677–684. 10.1016/j.molcel.2015.06.029 26236013 PMC4546541

[B36] Fujimori F. Takahashi K. Uchida C. Uchida T. (1999). Mice lacking Pin1 develop normally, but are defective in entering cell cycle from G(0) arrest. *Biochem. Biophys. Res. Commun.* 265 658–663. 10.1006/bbrc.1999.1736 10600477

[B37] Fujimoto Y. Shiraki T. Horiuchi Y. Waku T. Shigenaga A. Otaka A. (2010). Proline cis/trans-isomerase Pin1 regulates peroxisome proliferator-activated receptor gamma activity through the direct binding to the activation function-1 domain. *J. Biol. Chem.* 285 3126–3132. 10.1074/jbc.m109.055095 19996102 PMC2823398

[B38] Fujinaga K. Irwin D. Huang Y. Taube R. Kurosu T. Peterlin B. M. (2004). Dynamics of human immunodeficiency virus transcription: P-TEFb phosphorylates RD and dissociates negative effectors from the transactivation response element. *Mol. Cell. Biol.* 24 787–795. 10.1128/mcb.24.2.787-795.2004 14701750 PMC343783

[B39] Ghosh A. Shuman S. Lima C. D. (2011). Structural insights to how mammalian capping enzyme reads the CTD code. *Mol. Cell* 43 299–310. 10.1016/j.molcel.2011.06.001 21683636 PMC3142331

[B40] Gianni M. Boldetti A. Guarnaccia V. Rambaldi A. Parrella E. Raska I. (2009). Inhibition of the peptidyl-prolyl-isomerase Pin1 enhances the responses of acute myeloid leukemia cells to retinoic acid via stabilization of RARalpha and PML-RARalpha. *Cancer Res.* 69 1016–1026. 10.1158/0008-5472.can-08-2603 19155306

[B41] Ha M. Kim V. N. (2014). Regulation of microRNA biogenesis. *Nat. Rev. Mol. Cell Biol.* 15 509–524. 25027649 10.1038/nrm3838

[B42] Hahn S. (2004). Structure and mechanism of the RNA polymerase II transcription machinery. *Nat. Struct. Mol. Biol.* 11 394–403. 10.1038/nsmb763 15114340 PMC1189732

[B43] Halees A. S. El-Badrawi R. Khabar K. S. (2008). ARED organism: expansion of ARED reveals AU-rich element cluster variations between human and mouse. *Nucleic Acids Res.* 36 D137–D140. 17984078 10.1093/nar/gkm959 PMC2238997

[B44] Han H. J. Kwon N. Choi M. A. Jung K. O. Piao J. Y. Ngo H. K. (2016). Peptidyl prolyl isomerase PIN1 directly binds to and stabilizes hypoxia-inducible factor-1alpha. *PLoS ONE* 11:e0147038. 10.1371/journal.pone.0147038 26784107 PMC4718546

[B45] Han Y. Lee S. H. Bahn M. Yeo C. Y. Lee K. Y. (2016). Pin1 enhances adipocyte differentiation by positively regulating the transcriptional activity of PPARgamma. *Mol. Cell Endocrinol.* 436 150–158. 10.1016/j.mce.2016.07.030 27475846

[B46] Hanes S. D. (2015). Prolyl isomerases in gene transcription. *Biochim. Biophys. Acta* 1850 2017–2034. 10.1016/j.bbagen.2014.10.028 25450176 PMC4417086

[B47] Heintz N. Sive H. L. Roeder R. G. (1983). Regulation of human histone gene expression: kinetics of accumulation and changes in the rate of synthesis and in the half-lives of individual histone mRNAs during the HeLa cell cycle. *Mol. Cell. Biol.* 3 539–550. 10.1128/mcb.3.4.539 6406835 PMC368569

[B48] Henderson B. R. (2000). Nuclear-cytoplasmic shuttling of APC regulates beta-catenin subcellular localization and turnover. *Nat. Cell Biol.* 2 653–660. 10.1038/35023605 10980707

[B49] Hensel Z. Xiao J. (2013). Single-molecule methods for studying gene regulation in vivo. *Pflugers. Arch.* 465 383–395. 10.1007/s00424-013-1243-y 23430319 PMC3595547

[B50] Hohmann P. (1983). Phosphorylation of H1 histones. *Mol. Cell. Biochem.* 57 81–92. 6358859 10.1007/BF00223526

[B51] Hsin J. P. Manley J. L. (2012). The RNA polymerase II CTD coordinates transcription and RNA processing. *Genes Dev.* 26 2119–2137. 10.1101/gad.200303.112 23028141 PMC3465734

[B52] Hu X. Dong S. H. Chen J. Zhou X. Z. Chen R. Nair S. (2017). Prolyl isomerase PIN1 regulates the stability, transcriptional activity and oncogenic potential of BRD4. *Oncogene* 36 5177–5188. 10.1038/onc.2017.137 28481868 PMC5589477

[B53] Huang G. L. Liao D. Chen H. Lu Y. Chen L. Li H. (2016). The protein level and transcription activity of activating transcription factor 1 is regulated by prolyl isomerase Pin1 in nasopharyngeal carcinoma progression. *Cell Death Dis.* 7:e2571. 10.1038/cddis.2016.349 28032861 PMC5260992

[B54] Hunter T. Karin M. (1992). The regulation of transcription by phosphorylation. *Cell* 70 375–387. 10.1016/0092-8674(92)90162-6 1643656

[B55] Jacobs M. D. Harrison S. C. (1998). Structure of an IkappaBalpha/NF-kappaB complex. *Cell* 95 749–758. 9865693 10.1016/s0092-8674(00)81698-0

[B56] Jalouli M. Dery M. A. Lafleur V. N. Lamalice L. Zhou X. Z. Lu K. P. (2014). The prolyl isomerase Pin1 regulates hypoxia-inducible transcription factor (HIF) activity. *Cell. Signal.* 26 1649–1656. 10.1016/j.cellsig.2014.04.005 24726894

[B57] Jang M. K. Mochizuki K. Zhou M. Jeong H. S. Brady J. N. Ozato K. (2005). The bromodomain protein Brd4 is a positive regulatory component of P-TEFb and stimulates RNA polymerase II-dependent transcription. *Mol. Cell* 19 523–534. 10.1016/j.molcel.2005.06.027 16109376

[B58] Jung M. Gelato K. A. Fernandez-Montalvan A. Siegel S. Haendler B. (2015). Targeting BET bromodomains for cancer treatment. *Epigenomics* 7 487–501. 10.2217/epi.14.91 26077433

[B59] Komarnitsky P. Cho E. J. Buratowski S. (2000). Different phosphorylated forms of RNA polymerase II and associated mRNA processing factors during transcription. *Genes Dev.* 14 2452–2460. 10.1101/gad.824700 11018013 PMC316976

[B60] Kops O. Zhou X. Z. Lu K. P. (2002). Pin1 modulates the dephosphorylation of the RNA polymerase II C-terminal domain by yeast Fcp1. *FEBS Lett.* 513 305–311. 10.1016/s0014-5793(02)02288-3 11904169

[B61] Krishnan N. Lam T. T. Fritz A. Rempinski D. O’loughlin K. Minderman H. (2012). The prolyl isomerase Pin1 targets stem-loop binding protein (SLBP) to dissociate the SLBP-histone mRNA complex linking histone mRNA decay with SLBP ubiquitination. *Mol. Cell. Biol.* 32 4306–4322. 10.1128/mcb.00382-12 22907757 PMC3486140

[B62] Krishnan N. Titus M. A. Thapar R. (2014). The prolyl isomerase pin1 regulates mRNA levels of genes with short half-lives by targeting specific RNA binding proteins. *PLoS ONE* 9:e85427. 10.1371/journal.pone.0085427 24416409 PMC3887067

[B63] Kruiswijk F. Hasenfuss S. C. Sivapatham R. Baar M. P. Putavet D. Naipal K. A. (2016). Targeted inhibition of metastatic melanoma through interference with Pin1-FOXM1 signaling. *Oncogene* 35 2166–2177. 10.1038/onc.2015.282 26279295 PMC4757516

[B64] Kubicek K. Cerna H. Holub P. Pasulka J. Hrossova D. Loehr F. (2012). Serine phosphorylation and proline isomerization in RNAP II CTD control recruitment of Nrd1. *Genes Dev.* 26 1891–1896. 10.1101/gad.192781.112 22892239 PMC3435493

[B65] Lavoie S. B. Albert A. L. Handa H. Vincent M. Bensaude O. (2001). The peptidyl-prolyl isomerase Pin1 interacts with hSpt5 phosphorylated by Cdk9. *J. Mol. Biol.* 312 675–685. 10.1006/jmbi.2001.4991 11575923

[B66] Lee S. H. Choi Y. H. Kim Y. J. Choi H. S. Yeo C. Y. Lee K. Y. (2013). Prolyl isomerase Pin1 enhances osteoblast differentiation through Runx2 regulation. *FEBS Lett.* 587 3640–3647. 10.1016/j.febslet.2013.09.040 24113655

[B67] Lee S. H. Jeong H. M. Han Y. Cheong H. Kang B. Y. Lee K. Y. (2015). Prolyl isomerase Pin1 regulates the osteogenic activity of Osterix. *Mol. Cell. Endocrinol.* 400 32–40. 10.1016/j.mce.2014.11.017 25463757

[B68] Lee T. H. Chen C. H. Suizu F. Huang P. Schiene-Fischer C. Daum S. (2011). Death-associated protein kinase 1 phosphorylates Pin1 and inhibits its prolyl isomerase activity and cellular function. *Mol. Cell* 42 147–159. 10.1016/j.molcel.2011.03.005 21497122 PMC3088080

[B69] Li B. Carey M. Workman J. L. (2007). The role of chromatin during transcription. *Cell* 128 707–719. 10.1016/j.cell.2007.01.015 17320508

[B70] Li C. Chang D. L. Yang Z. Qi J. Liu R. He H. (2013). Pin1 modulates p63alpha protein stability in regulation of cell survival, proliferation and tumor formation. *Cell Death Dis.* 4:e943. 10.1038/cddis.2013.468 24309930 PMC3877541

[B71] Li J. Pu W. Sun H. L. Zhou J. K. Fan X. Zheng Y. (2018). Pin1 impairs microRNA biogenesis by mediating conformation change of XPO5 in hepatocellular carcinoma. *Cell Death. Differ.* 25 1612–1624. 10.1038/s41418-018-0065-z 29445125 PMC6143530

[B72] Li Y. Liu M. Chen L. F. Chen R. (2018). P-TEFb: finding its ways to release promoter-proximally paused RNA polymerase II. *Transcription* 9 88–94. 10.1080/21541264.2017.1281864 28102758 PMC5834220

[B73] Lian J. B. Stein G. S. (2003). Runx2/Cbfa1: a multifunctional regulator of bone formation. *Curr. Pharm. Des.* 9 2677–2685. 10.2174/1381612033453659 14529540

[B74] Liou Y. C. Ryo A. Huang H. K. Lu P. J. Bronson R. Fujimori F. (2002). Loss of Pin1 function in the mouse causes phenotypes resembling cyclin D1-null phenotypes. *Proc. Natl. Acad. Sci. U.S.A.* 99 1335–1340. 10.1073/pnas.032404099 11805292 PMC122191

[B75] Liou Y. C. Zhou X. Z. Lu K. P. (2011). Prolyl isomerase Pin1 as a molecular switch to determine the fate of phosphoproteins. *Trends Biochem. Sci.* 36 501–514. 10.1016/j.tibs.2011.07.001 21852138 PMC3185210

[B76] Liu W. Youn H. D. Zhou X. Z. Lu K. P. Liu J. O. (2001). Binding and regulation of the transcription factor NFAT by the peptidyl prolyl cis-trans isomerase Pin1. *FEBS Lett.* 496 105–108. 10.1016/s0014-5793(01)02411-5 11356192

[B77] Loflin P. Chen C. Y. Shyu A. B. (1999). Unraveling a cytoplasmic role for hnRNP D in the in vivo mRNA destabilization directed by the AU-rich element. *Genes Dev.* 13 1884–1897. 10.1101/gad.13.14.1884 10421639 PMC316883

[B78] Lu K. P. Zhou X. Z. (2007). The prolyl isomerase PIN1: a pivotal new twist in phosphorylation signalling and disease. *Nat. Rev. Mol. Cell Biol.* 8 904–916. 10.1038/nrm2261 17878917

[B79] Lufei C. Koh T. H. Uchida T. Cao X. (2007). Pin1 is required for the Ser727 phosphorylation-dependent Stat3 activity. *Oncogene* 26 7656–7664. 10.1038/sj.onc.1210567 17563747

[B80] Lunde B. M. Reichow S. L. Kim M. Suh H. Leeper T. C. Yang F. (2010). Cooperative interaction of transcription termination factors with the RNA polymerase II C-terminal domain. *Nat. Struct. Mol. Biol.* 17 1195–1201. 10.1038/nsmb.1893 20818393 PMC2950884

[B81] Lv L. Zhang J. Zhang L. Xue G. Wang P. Meng Q. (2013). Essential role of Pin1 *via* STAT3 signalling and mitochondria-dependent pathways in restenosis in type 2 diabetes. *J. Cell. Mol. Med.* 17 989–1005. 10.1111/jcmm.12082 23750710 PMC3780535

[B82] Lydon J. P. O’malley B. W. (2011). Minireview: steroid receptor coactivator-3: a multifarious coregulator in mammary gland metastasis. *Endocrinology* 152 19–25. 10.1210/en.2010-1012 21047941 PMC3219052

[B83] Magli A. Angelelli C. Ganassi M. Baruffaldi F. Matafora V. Battini R. (2010). Proline isomerase Pin1 represses terminal differentiation and myocyte enhancer factor 2C function in skeletal muscle cells. *J. Biol. Chem.* 285 34518–34527. 10.1074/jbc.m110.104133 20801874 PMC2966067

[B84] Mantovani F. Piazza S. Gostissa M. Strano S. Zacchi P. Mantovani R. (2004). Pin1 links the activities of c-Abl and p300 in regulating p73 function. *Mol. Cell* 14 625–636. 10.1016/j.molcel.2004.05.007 15175157

[B85] Mantovani F. Zannini A. Rustighi A. Del Sal G. (2015). Interaction of p53 with prolyl isomerases: healthy and unhealthy relationships. *Biochim. Biophys. Acta* 1850 2048–2060. 10.1016/j.bbagen.2015.01.013 25641576

[B86] Monje P. Hernandez-Losa J. Lyons R. J. Castellone M. D. Gutkind J. S. (2005). Regulation of the transcriptional activity of c-Fos by ERK. A novel role for the prolyl isomerase PIN1. *J. Biol. Chem.* 280 35081–35084. 10.1074/jbc.c500353200 16123044

[B87] Monje P. Marinissen M. J. Gutkind J. S. (2003). Phosphorylation of the carboxyl-terminal transactivation domain of c-Fos by extracellular signal-regulated kinase mediates the transcriptional activation of AP-1 and cellular transformation induced by platelet-derived growth factor. *Mol. Cell. Biol.* 23 7030–7043. 10.1128/mcb.23.19.7030-7043.2003 12972619 PMC193921

[B88] Moretto-Zita M. Jin H. Shen Z. Zhao T. Briggs S. P. Xu Y. (2010). Phosphorylation stabilizes Nanog by promoting its interaction with Pin1. *Proc. Natl. Acad. Sci. U.S.A.* 107 13312–13317. 10.1073/pnas.1005847107 20622153 PMC2922169

[B89] Muller S. Filippakopoulos P. Knapp S. (2011). Bromodomains as therapeutic targets. *Expert Rev. Mol. Med.* 13 e29. 10.1017/S1462399411001992 PMC3177561 21933453

[B90] Nag S. Qin J. Srivenugopal K. S. Wang M. Zhang R. (2013). The MDM2-p53 pathway revisited. *J. Biomed. Res.* 27 254–271. 23885265 10.7555/JBR.27.20130030 PMC3721034

[B91] Nakada S. Kuboki S. Nojima H. Yoshitomi H. Furukawa K. Takayashiki T. (2019). Roles of Pin1 as a key molecule for EMT induction by activation of STAT3 and NF-κB in human gallbladder cancer. *Ann. Surg. Oncol.* 26 907–917. 10.1245/s10434-018-07132-7 30610554

[B92] Nakamura K. Kosugi I. Lee D. Y. Hafner A. Sinclair D. A. Ryo A. (2012). Prolyl isomerase Pin1 regulates neuronal differentiation via beta-catenin. *Mol. Cell. Biol.* 32 2966–2978. 10.1128/mcb.05688-11 22645310 PMC3434519

[B93] Nakano A. Koinuma D. Miyazawa K. Uchida T. Saitoh M. Kawabata M. (2009). Pin1 down-regulates transforming growth factor-beta (TGF-beta) signaling by inducing degradation of Smad proteins. *J. Biol. Chem.* 284 6109–6115. 10.1074/jbc.m804659200 19122240

[B94] Nakatsu Y. Matsunaga Y. Ueda K. Yamamotoya T. Inoue Y. Inoue M. K. (2018). Development of Pin1 inhibitors and their potential as therapeutic agents. *Curr. Med. Chem* 10.2174/0929867325666181105120911 [Epub ahead of print]. 30394205

[B95] Nakatsu Y. Matsunaga Y. Yamamotoya T. Ueda K. Inoue M. K. Mizuno Y. (2019). Prolyl isomerase Pin1 suppresses thermogenic programs in adipocytes by promoting degradation of transcriptional co-activator PRDM16. *Cell Rep.* 26:e3223. 10.1016/j.celrep.2019.02.066 30893596

[B96] Nakatsu Y. Matsunaga Y. Yamamotoya T. Ueda K. Inoue Y. Mori K. (2016). Physiological and pathogenic roles of prolyl isomerase pin1 in metabolic regulations via multiple signal transduction pathway modulations. *Int. J. Mol. Sci.* 17:1495. 10.3390/ijms17091495 27618008 PMC5037772

[B97] Nakatsu Y. Sakoda H. Kushiyama A. Ono H. Fujishiro M. Horike N. (2010). Pin1 associates with and induces translocation of CRTC2 to the cytosol, thereby suppressing cAMP-responsive element transcriptional activity. *J. Biol. Chem.* 285 33018–33027. 10.1074/jbc.m110.137836 20675384 PMC2963389

[B98] Nechama M. Ben-Dov I. Z. Briata P. Gherzi R. Naveh-Many T. (2008). The mRNA decay promoting factor K-homology splicing regulator protein post-transcriptionally determines parathyroid hormone mRNA levels. *FASEB J.* 22 3458–3468. 10.1096/fj.08-107250 18583400

[B99] Nechama M. Uchida T. Mor Yosef-Levi I. Silver J. Naveh-Many T. (2009). The peptidyl-prolyl isomerase Pin1 determines parathyroid hormone mRNA levels and stability in rat models of secondary hyperparathyroidism. *J. Clin. Invest.* 119 3102–3114. 10.1172/jci39522 19770516 PMC2752082

[B100] Nicole Tsang Y. H. Wu X. W. Lim J. S. Wee Ong C. Salto-Tellez M. Ito K. (2013). Prolyl isomerase Pin1 downregulates tumor suppressor RUNX3 in breast cancer. *Oncogene* 32 1488–1496. 10.1038/onc.2012.178 22580604 PMC3438320

[B101] Nishi M. Akutsu H. Masui S. Kondo A. Nagashima Y. Kimura H. (2011). A distinct role for Pin1 in the induction and maintenance of pluripotency. *J. Biol. Chem.* 286 11593–11603. 10.1074/jbc.m110.187989 21296877 PMC3064213

[B102] Noble C. G. Hollingworth D. Martin S. R. Ennis-Adeniran V. Smerdon S. J. Kelly G. (2005). Key features of the interaction between Pcf11 CID and RNA polymerase II CTD. *Nat. Struct. Mol. Biol.* 12 144–151. 10.1038/nsmb887 15665873

[B103] Patikoglou G. Burley S. K. (1997). Eukaryotic transcription factor-DNA complexes. *Annu. Rev. Biophys. Biomol. Struct.* 26 289–325. 10.1146/annurev.biophys.26.1.289 9241421

[B104] Pawson T. Scott J. D. (2005). Protein phosphorylation in signaling–50 years and counting. *Trends Biochem. Sci.* 30 286–290. 10.1016/j.tibs.2005.04.013 15950870

[B105] Phatnani H. P. Greenleaf A. L. (2006). Phosphorylation and functions of the RNA polymerase II CTD. *Genes Dev.* 20 2922–2936. 10.1101/gad.1477006 17079683

[B106] Poolman T. M. Farrow S. N. Matthews L. Loudon A. S. Ray D. W. (2013). Pin1 promotes GR transactivation by enhancing recruitment to target genes. *Nucleic Acids Res.* 41 8515–8525. 10.1093/nar/gkt624 23887939 PMC3794586

[B107] Pu W. Li J. Zheng Y. Shen X. Fan X. Zhou J. K. (2018). Targeting Pin1 by inhibitor API-1 regulates microRNA biogenesis and suppresses hepatocellular carcinoma development. *Hepatology* 68 547–560. 10.1002/hep.29819 29381806

[B108] Pulikkan J. A. Dengler V. Peer Zada A. A. Kawasaki A. Geletu M. Pasalic Z. (2010). Elevated PIN1 expression by C/EBPalpha-p30 blocks C/EBPalpha-induced granulocytic differentiation through c-Jun in AML. *Leukemia* 24 914–923. 10.1038/leu.2010.37 20376080 PMC2923485

[B109] Raghuram N. Strickfaden H. Mcdonald D. Williams K. Fang H. Mizzen C. (2013). Pin1 promotes histone H1 dephosphorylation and stabilizes its binding to chromatin. *J. Cell Biol.* 203 57–71. 10.1083/jcb.201305159 24100296 PMC3798258

[B110] Rajbhandari P. Finn G. Solodin N. M. Singarapu K. K. Sahu S. C. Markley J. L. (2012). Regulation of estrogen receptor alpha N-terminus conformation and function by peptidyl prolyl isomerase Pin1. *Mol. Cell. Biol.* 32 445–457. 10.1128/mcb.06073-11 22064478 PMC3255769

[B111] Rajbhandari P. Ozers M. S. Solodin N. M. Warren C. L. Alarid E. T. (2015). Peptidylprolyl isomerase pin1 directly enhances the DNA binding functions of estrogen receptor alpha. *J. Biol. Chem.* 290 13749–13762. 10.1074/jbc.m114.621698 25866209 PMC4447953

[B112] Rajbhandari P. Schalper K. A. Solodin N. M. Ellison-Zelski S. J. Ping, Lu K. (2014). Pin1 modulates ERalpha levels in breast cancer through inhibition of phosphorylation-dependent ubiquitination and degradation. *Oncogene* 33 1438–1447. 10.1038/onc.2013.78 23542176 PMC3815749

[B113] Rangasamy V. Mishra R. Sondarva G. Das S. Lee T. H. Bakowska J. C. (2012). Mixed-lineage kinase 3 phosphorylates prolyl-isomerase Pin1 to regulate its nuclear translocation and cellular function. *Proc. Natl. Acad. Sci. U.S.A.* 109 8149–8154. 10.1073/pnas.1200804109 22566623 PMC3361382

[B114] Restelli M. Lopardo T. Lo Iacono N. Garaffo G. Conte D. Rustighi A. (2014). DLX5, FGF8 and the Pin1 isomerase control DeltaNp63alpha protein stability during limb development: a regulatory loop at the basis of the SHFM and EEC congenital malformations. *Hum. Mol. Genet.* 23 3830–3842. 10.1093/hmg/ddu096 24569166 PMC4065156

[B115] Richard P. Manley J. L. (2009). Transcription termination by nuclear RNA polymerases. *Genes Dev.* 23 1247–1269. 10.1101/gad.1792809 19487567 PMC2763537

[B116] Ross D. A. Kadesch T. (2001). The notch intracellular domain can function as a coactivator for LEF-1. *Mol. Cell. Biol.* 21 7537–7544. 10.1128/mcb.21.22.7537-7544.2001 11604490 PMC99925

[B117] Rustighi A. Tiberi L. Soldano A. Napoli M. Nuciforo P. Rosato A. (2009). The prolyl-isomerase Pin1 is a Notch1 target that enhances Notch1 activation in cancer. *Nat. Cell Biol.* 11 133–142. 10.1038/ncb1822 19151708

[B118] Ryo A. Nakamura M. Wulf G. Liou Y. C. Lu K. P. (2001). Pin1 regulates turnover and subcellular localization of beta-catenin by inhibiting its interaction with APC. *Nat. Cell Biol.* 3 793–801. 10.1038/ncb0901-793 11533658

[B119] Ryo A. Suizu F. Yoshida Y. Perrem K. Liou Y. C. Wulf G. (2003). Regulation of NF-kappaB signaling by Pin1-dependent prolyl isomerization and ubiquitin-mediated proteolysis of p65/RelA. *Mol. Cell* 12 1413–1426. 10.1016/s1097-2765(03)00490-8 14690596

[B120] Saitoh T. Tun-Kyi A. Ryo A. Yamamoto M. Finn G. Fujita T. (2006). Negative regulation of interferon-regulatory factor 3-dependent innate antiviral response by the prolyl isomerase Pin1. *Nat. Immunol.* 7 598–605. 10.1038/ni1347 16699525

[B121] Sanchez-Arevalo Lobo V. J. Doni M. Verrecchia A. Sanulli S. Faga G. Piontini A. (2013). Dual regulation of Myc by Abl. *Oncogene* 32 5261–5271. 10.1038/onc.2012.621 23318434 PMC3914638

[B122] Schoenberg D. R. Maquat L. E. (2012). Regulation of cytoplasmic mRNA decay. *Nat. Rev. Genet.* 13 246–259. 10.1038/nrg3160 22392217 PMC3351101

[B123] Segil N. Guermah M. Hoffmann A. Roeder R. G. Heintz N. (1996). Mitotic regulation of TFIID: inhibition of activator-dependent transcription and changes in subcellular localization. *Genes Dev.* 10 2389–2400. 10.1101/gad.10.19.2389 8843192

[B124] Shaw P. E. (2007). Peptidyl-prolyl cis/trans isomerases and transcription: is there a twist in the tail? *EMBO Rep.* 8 40–45. 10.1038/sj.embor.7400873 17203101 PMC1796747

[B125] Shen Z. J. Esnault S. Malter J. S. (2005). The peptidyl-prolyl isomerase Pin1 regulates the stability of granulocyte-macrophage colony-stimulating factor mRNA in activated eosinophils. *Nat. Immunol.* 6 1280–1287. 10.1038/ni1266 16273101

[B126] Shen Z. J. Esnault S. Rosenthal L. A. Szakaly R. J. Sorkness R. L. Westmark P. R. (2008). Pin1 regulates TGF-beta1 production by activated human and murine eosinophils and contributes to allergic lung fibrosis. *J. Clin. Invest.* 118 479–490. 18188456 10.1172/JCI32789 PMC2176187

[B127] Shen Z. J. Malter J. S. (2015). Regulation of AU-Rich element RNA binding proteins by phosphorylation and the prolyl isomerase Pin1. *Biomolecules* 5 412–434. 10.3390/biom5020412 25874604 PMC4496679

[B128] Shimizu T. Bamba Y. Kawabe Y. Fukuda T. Fujimori F. Takahashi K. (2016). Prolyl isomerase Pin1 regulates doxorubicin-inducible P-glycoprotein level by reducing Foxo3 stability. *Biochem. Biophys. Res. Commun.* 471 328–333. 10.1016/j.bbrc.2016.02.014 26874277

[B129] Shin H. R. Islam R. Yoon W. J. Lee T. Cho Y. D. Bae H. S. (2016). Pin1-mediated modification prolongs the nuclear retention of beta-catenin in Wnt3a-induced osteoblast differentiation. *J. Biol. Chem.* 291 5555–5565. 10.1074/jbc.m115.698563 26740630 PMC4786698

[B130] Shinoda K. Kuboki S. Shimizu H. Ohtsuka M. Kato A. Yoshitomi H. (2015). Pin1 facilitates NF-kappaB activation and promotes tumour progression in human hepatocellular carcinoma. *Br. J. Cancer* 113 1323–1331. 10.1038/bjc.2015.272 26461058 PMC4815797

[B131] Sogaard T. M. Svejstrup J. Q. (2007). Hyperphosphorylation of the C-terminal repeat domain of RNA polymerase II facilitates dissociation of its complex with mediator. *J. Biol. Chem.* 282 14113–14120. 10.1074/jbc.m701345200 17376774

[B132] Spiegelman B. M. Heinrich R. (2004). Biological control through regulated transcriptional coactivators. *Cell* 119 157–167. 10.1016/j.cell.2004.09.037 15479634

[B133] Splinter E. De Laat W. (2011). The complex transcription regulatory landscape of our genome: control in three dimensions. *EMBO J.* 30 4345–4355. 10.1038/emboj.2011.344 21952046 PMC3230377

[B134] Srivastava R. Ahn S. H. (2015). Modifications of RNA polymerase II CTD: connections to the histone code and cellular function. *Biotechnol. Adv.* 33 856–872. 10.1016/j.biotechadv.2015.07.008 26241863

[B135] Stanya K. J. Kao H. Y. (2009). New insights into the functions and regulation of the transcriptional corepressors SMRT and N-CoR. *Cell Div.* 4:7. 10.1186/1747-1028-4-7 19383165 PMC2678994

[B136] Stanya K. J. Liu Y. Means A. R. Kao H. Y. (2008). Cdk2 and Pin1 negatively regulate the transcriptional corepressor SMRT. *J. Cell Biol.* 183 49–61. 10.1083/jcb.200806172 18838553 PMC2557042

[B137] Thomas M. C. Chiang C. M. (2006). The general transcription machinery and general cofactors. *Crit. Rev. Biochem. Mol. Biol* 41 105–178. 10.1080/10409230600648736 16858867

[B138] Van Der Horst A. De Vries-Smits A. M. Brenkman A. B. Van Triest M. H. Van Den Broek N. Colland F. (2006). FOXO4 transcriptional activity is regulated by monoubiquitination and USP7/HAUSP. *Nat. Cell Biol.* 8 1064–1073. 10.1038/ncb1469 16964248

[B139] van Tiel C. M. Kurakula K. Koenis D. S. van der Wal E. de Vries C. J. (2012). Dual function of Pin1 in NR4A nuclear receptor activation: enhanced activity of NR4As and increased Nur77 protein stability. *Biochim. Biophys. Acta* 1823 1894–1904. 10.1016/j.bbamcr.2012.06.030 22789442

[B140] Vicent G. P. Nacht A. S. Font-Mateu J. Castellano G. Gaveglia L. Ballare C. (2011). Four enzymes cooperate to displace histone H1 during the first minute of hormonal gene activation. *Genes Dev.* 25 845–862. 10.1101/gad.621811 21447625 PMC3078709

[B141] Wada T. Takagi T. Yamaguchi Y. Ferdous A. Imai T. Hirose S. (1998). DSIF, a novel transcription elongation factor that regulates RNA polymerase II processivity, is composed of human Spt4 and Spt5 homologs. *Genes Dev.* 12 343–356. 10.1101/gad.12.3.343 9450929 PMC316480

[B142] Wang T. Liu Z. Shi F. Wang J. (2016). Pin1 modulates chemo-resistance by up-regulating FoxM1 and the involvements of Wnt/beta-catenin signaling pathway in cervical cancer. *Mol. Cell. Biochem.* 413 179–187. 10.1007/s11010-015-2651-4 26820938

[B143] Wang Z. F. Whitfield M. L. Ingledue T. C. III Dominski Z. Marzluff W. F. (1996). The protein that binds the 3′ end of histone mRNA: a novel RNA-binding protein required for histone pre-mRNA processing. *Genes Dev.* 10 3028–3040. 10.1101/gad.10.23.3028 8957003

[B144] Werner-Allen J. W. Lee C. J. Liu P. Nicely N. I. Wang S. Greenleaf A. L. (2011). cis-Proline-mediated Ser(P)5 dephosphorylation by the RNA polymerase II C-terminal domain phosphatase Ssu72. *J. Biol. Chem.* 286 5717–5726. 10.1074/jbc.m110.197129 21159777 PMC3037684

[B145] Wu X. Qi J. Bradner J. E. Xiao G. Chen L. F. (2013). Bromodomain and extraterminal (BET) protein inhibition suppresses human T cell leukemia virus 1 (HTLV-1) Tax protein-mediated tumorigenesis by inhibiting nuclear factor kappaB (NF-kappaB) signaling. *J. Biol. Chem.* 288 36094–36105. 10.1074/jbc.m113.485029 24189064 PMC3861657

[B146] Wulf G. M. Liou Y. C. Ryo A. Lee S. W. Lu K. P. (2002). Role of Pin1 in the regulation of p53 stability and p21 transactivation, and cell cycle checkpoints in response to DNA damage. *J. Biol. Chem.* 277 47976–47979. 10.1074/jbc.c200538200 12388558

[B147] Wulf G. M. Ryo A. Wulf G. G. Lee S. W. Niu T. Petkova V. (2001). Pin1 is overexpressed in breast cancer and cooperates with Ras signaling in increasing the transcriptional activity of c-Jun towards cyclin D1. *EMBO J.* 20 3459–3472. 10.1093/emboj/20.13.3459 11432833 PMC125530

[B148] Xiang K. Nagaike T. Xiang S. Kilic T. Beh M. M. Manley J. L. (2010). Crystal structure of the human symplekin-Ssu72-CTD phosphopeptide complex. *Nature* 467 729–733. 10.1038/nature09391 20861839 PMC3038789

[B149] Xu Y. X. Hirose Y. Zhou X. Z. Lu K. P. Manley J. L. (2003). Pin1 modulates the structure and function of human RNA polymerase II. *Genes Dev.* 17 2765–2776. 10.1101/gad.1135503 14600023 PMC280625

[B150] Xu Y. X. Manley J. L. (2007a). Pin1 modulates RNA polymerase II activity during the transcription cycle. *Genes Dev.* 21 2950–2962. 10.1101/gad.1592807 18006688 PMC2049196

[B151] Xu Y. X. Manley J. L. (2007b). The prolyl isomerase Pin1 functions in mitotic chromosome condensation. *Mol. Cell* 26 287–300. 10.1016/j.molcel.2007.03.020 17466629

[B152] Yamaguchi Y. Takagi T. Wada T. Yano K. Furuya A. Sugimoto S. (1999). NELF, a multisubunit complex containing RD, cooperates with DSIF to repress RNA polymerase II elongation. *Cell* 97 41–51. 10.1016/s0092-8674(00)80713-8 10199401

[B153] Yang H. C. Chuang J. Y. Jeng W. Y. Liu C. I. Wang A. H. Lu P. J. (2014). Pin1-mediated Sp1 phosphorylation by CDK1 increases Sp1 stability and decreases its DNA-binding activity during mitosis. *Nucleic Acids Res.* 42 13573–13587. 10.1093/nar/gku1145 25398907 PMC4267622

[B154] Yeh E. Cunningham M. Arnold H. Chasse D. Monteith T. Ivaldi G. (2004). A signalling pathway controlling c-Myc degradation that impacts oncogenic transformation of human cells. *Nat. Cell Biol.* 6 308–318. 10.1038/ncb1110 15048125

[B155] Yi P. Wu R. C. Sandquist J. Wong J. Tsai S. Y. Tsai M. J. (2005). Peptidyl-prolyl isomerase 1 (Pin1) serves as a coactivator of steroid receptor by regulating the activity of phosphorylated steroid receptor coactivator 3 (SRC-3/AIB1). *Mol. Cell. Biol.* 25 9687–9699. 10.1128/mcb.25.21.9687-9699.2005 16227615 PMC1265806

[B156] Yoon W. J. Islam R. Cho Y. D. Woo K. M. Baek J. H. Uchida T. (2013). Pin1-mediated Runx2 modification is critical for skeletal development. *J. Cell. Physiol.* 228 2377–2385. 10.1002/jcp.24403 23702614 PMC4051422

[B157] Zacchi P. Gostissa M. Uchida T. Salvagno C. Avolio F. Volinia S. (2002). The prolyl isomerase Pin1 reveals a mechanism to control p53 functions after genotoxic insults. *Nature* 419 853–857. 10.1038/nature01120 12397362

[B158] Zannini A. Rustighi A. Campaner E. Del Sal G. (2019). Oncogenic hijacking of the PIN1 signaling network. *Front. Oncol.* 9:94. 10.3389/fonc.2019.00094 30873382 PMC6401644

[B159] Zhang J. Chen M. Zhu Y. Dai X. Dang F. Ren J. (2019). SPOP promotes nanog destruction to suppress stem cell traits and prostate cancer progression. *Dev. Cell* 48:e325. 10.1016/j.devcel.2018.11.035 PMC6462403 30595538

[B160] Zhang M. Wang X. J. Chen X. Bowman M. E. Luo Y. Noel J. P. (2012). Structural and kinetic analysis of prolyl-isomerization/phosphorylation cross-talk in the CTD code. *ACS Chem. Biol.* 7 1462–1470. 10.1021/cb3000887 22670809 PMC3423551

[B161] Zhang Y. Kim Y. Genoud N. Gao J. Kelly J. W. Pfaff S. L. (2006). Determinants for dephosphorylation of the RNA polymerase II C-terminal domain by Scp1. *Mol. Cell* 24 759–770. 10.1016/j.molcel.2006.10.027 17157258 PMC2859291

[B162] Zheng H. You H. Zhou X. Z. Murray S. A. Uchida T. Wulf G. (2002). The prolyl isomerase Pin1 is a regulator of p53 in genotoxic response. *Nature* 419 849–853. 10.1038/nature01116 12397361

[B163] Zhou X. Z. Lu K. P. (2016). The isomerase PIN1 controls numerous cancer-driving pathways and is a unique drug target. *Nat. Rev. Cancer* 16 463–478. 10.1038/nrc.2016.49 27256007

[B164] Zhu J. Mckeon F. (2000). Nucleocytoplasmic shuttling and the control of NF-AT signaling. *Cell Mol. Life Sci.* 57 411–420. 10.1007/pl00000703 10823242 PMC11147004

[B165] Zilfou J. T. Lowe S. W. (2009). Tumor suppressive functions of p53. *Cold Spring Harb. Perspect. Biol.* 1:a001883. 10.1101/cshperspect.a001883 20066118 PMC2773645

